# Supramolecular delivery of dinuclear ruthenium and osmium MCU inhibitors†

**DOI:** 10.1039/d4qi01102c

**Published:** 2024-07-11

**Authors:** Nicholas P. Bigham, Robyn J. Novorolsky, Keana R. Davis, Haipei Zou, Samantha N. MacMillan, Michael J. Stevenson, George S. Robertson, Justin J. Wilson

**Affiliations:** a Department of Chemistry and Chemical Biology, Cornell University Ithaca NY 14853 USA jjw275@cornell.edu; b Department of Pharmacology, Faculty of Medicine, Dalhousie University 6th Floor Sir Charles Tupper Medical Building Halifax B3H 4R2 Canada; c Brain Repair Centre, Faculty of Medicine, Dalhousie University, Life Sciences Research Institute Halifax NS B3H 4R2 Canada; d Department of Psychiatry, Faculty of Medicine, Dalhousie University Halifax NS B3H 2E2 Canada; e Department of Chemistry, University of San Francisco San Francisco CA 94117 USA; f Department of Chemistry & Biochemistry, University of California Santa Barbara Santa Barbara CA 93106 USA

## Abstract

The transmembrane protein known as the mitochondrial calcium uniporter (MCU) mediates the influx of calcium ions (Ca^2+^) into the mitochondrial matrix. An overload of mitochondrial Ca^2+^ (_*m*_Ca^2+^) is directly linked to damaging effects in pathological conditions. Therefore, inhibitors of the MCU are important chemical biology tools and therapeutic agents. Here, two new analogues of previously reported Ru- and Os-based MCU inhibitors Ru265 and Os245, of the general formula [(C_10_H_15_CO_2_)M(NH_3_)_4_(μ-N)M(NH_3_)_4_(O_2_CC_10_H_15_)](CF_3_SO_3_)_3_, where M = Ru (1) or Os (2), are reported. These analogues bear adamantane functional groups, which were installed to act as guests for the host molecule cucurbit-[7]-uril (CB[7]). These complexes were characterized and analyzed for their efficiency as guests for CB[7]. As shown through a variety of spectroscopic techniques, each adamantane ligand is encapsulated into one CB[7], affording a supramolecular complex of 1 : 2 stoichiometry. The biological effects of these compounds in the presence and absence of two equiv. CB[7] were assessed. Both complexes 1 and 2 exhibit enhanced cellular uptake compared to the parent compounds Ru265 and Os245, and their uptake is increased further in the presence of CB[7]. Compared to Ru265 and Os245, 1 and 2 are less potent as _*m*_Ca^2+^ uptake inhibitors in permeabilized cell models. However, in intact cell systems, 1 and 2 inhibit the MCU at concentrations as low as 1 μM, marking an advantage over Ru265 and Os245 which require an order of magnitude higher doses for similar biological effects. The presence of CB[7] did not affect the inhibitory properties of 1 and 2. Experiments in primary cortical neurons showed that 1 and 2 can elicit protective effects against oxygen-glucose deprivation at lower doses than those required for Ru265 or Os245. At low concentrations, the protective effects of 1 were modulated by CB[7], suggesting that supramolecular complex formation can play a role in these biological conditions. The *in vivo* biocompatibility of 1 was investigated in mice. The intraperitoneal administration of these compounds and their CB[7] complexes led to time-dependent induction of seizures with no protective effects elicited by CB[7]. This work demonstrates the potential for supramolecular interactions in the development of MCU inhibitors.

## Abstract 2

The mitochondrial calcium uniporter (MCU) inhibitors Ru265 and Os245 were functionalized with adamantane to make them amenable for encapsulation in cucurbituril. The resulting host–guest complexes were investigated for their biological properties.

## Graphical abstract

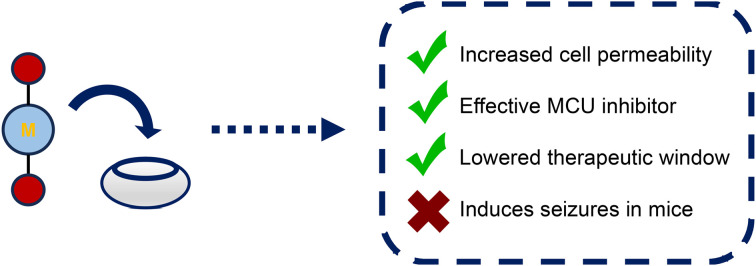

**10th anniversary statement**

Prior to joining Inorganic Chemistry Frontiers as an associate editor in 2023, my literature searches within bioinorganic medicinal chemistry would often lead me to this new journal. I was impressed by the high-quality of the manuscripts within this field, as well as their increasing number and visibility. This new journal was arising to a top-tier level. I was a consistent reviewer for this journal, and I saw many interesting and high-impact studies. Now, as an associate editor, I have been pleased to see the submission of such manuscripts first-hand. In all of my roles at Inorganic Chemistry Frontiers as a reader, reviewer, author, and editor, I have been impressed and excited about the science within this journal. In particular, I think it has arisen as a fantastic forum to disseminate studies in bioinorganic chemistry to the broader community.

## Introduction

The biochemistry of calcium ions (Ca^2+^) has been widely studied due to its importance in cellular function and signaling.^1–3^ Notably, the mitochondria are critical for regulating intracellular Ca^2+^ levels, and mitochondrial Ca^2+^ (_*m*_Ca^2+^) uptake is an important process for maintaining cellular homeostasis.^4–6^ The dysregulation of _*m*_Ca^2+^ levels, however, can lead to damaging effects and pathological conditions. Most prominently, excessive levels of Ca^2+^ in the mitochondria lead to _*m*_Ca^2+^ overload, which triggers cell death.^7,8^ The phenomenon of _*m*_Ca^2+^ overload and its damaging effects have been implicated in a number of pathological conditions such as heart disease,^9–11^ cancer,^12,13^ cystic fibrosis,^14,15^ neurodegenerative disorders,^16,17^ and ischemia-reperfusion injury.^18–20^ Given the physiological importance of _*m*_Ca^2+^ levels, substantial research has been committed to understand how the mitochondria regulate Ca^2+^ uptake. These efforts have revealed the mitochondrial calcium uniporter (MCU) complex to be responsible for mediating Ca^2+^ uptake into this organelle.^6,21–24^ This tetrameric, transmembrane protein consists of the pore-forming MCU subunit^25–28^ and three regulatory subunits – EMRE,^29^ MICU1,^30^ and MICU2.^31^ Both MICU1 and MICU2 contain EF-hand domains to sense extramitochondrial Ca^2+^ levels and interact with the MCU subunit at a highly conserved solvent-exposed DXXE motif near the pore opening.^25,26^ Upon exposure to high cytosolic Ca^2+^ concentrations, these EF-hand domains dissociate from the MCU subunit,^31–34^ allowing for Ca^2+^ to traverse the pore and enter the mitochondrial matrix.^35,36^ Therefore, targeting the DXXE motif with chemical inhibitors could be a viable approach to prevent _*m*_Ca^2+^ overload as a potential therapeutic strategy.

**Chart 1:**
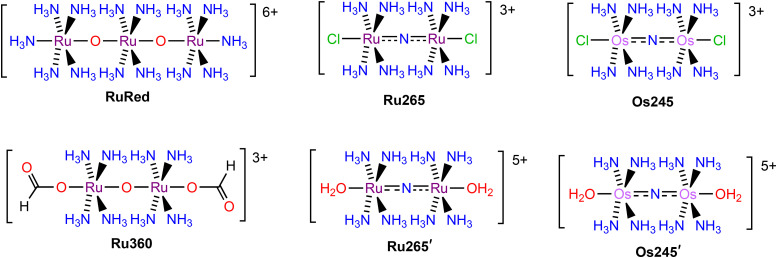
Chemical structures of ruthenium and osmium MCU inhibitors.

A number of organic^37–42^ and inorganic^43–50^ inhibitors of the MCU have been reported to date. The most well-known and widely used MCU inhibitors are the polynuclear Ru complexes, ruthenium red (RuRed) and Ru360 (Chart 1). Subsequent work on this compound class led to the discovery of the dinuclear nitrido-bridged Ru compound named Ru265 (Chart 1).^47^ In contrast to the earlier RuRed and Ru360, this complex, which exhibits nanomolar MCU-inhibitory activity, is cell permeable and stable towards biological reduction,^51,52^ properties that make it a useful tool for intact cell systems. Given the significant promise of Ru265 as an MCU inhibitor, our group has developed analogues in order to understand structure–activity relationships for this compound class and to identify those with improved properties for biological applications. A key observation is that the axial chloride ligands of Ru265 dissociate within a timescale of minutes under physiological conditions to give the aquated complex Ru265′ (Chart 1), which itself is a potent MCU inhibitor.^51^ Notably, replacing these chlorides with carboxylates substantially slows the aquation process. Careful modification of the carboxylates has conferred these compounds with prodrug capabilities,^53^ higher cell uptake,^54^ and fluorogenic properties.^55^ In addition, we have reported and studied the MCU-inhibitory properties of the Os-analogue of Ru265, named Os245 (Chart 1).^50^ The aquation of the axial chloride ligands of this complex to form Os245′ (Chart 1) occurs at a rate nearly 100× slower than that of Ru265. The slower axial chloride substitution rate of Os245 is consistent with the known greater kinetic inertness of 5d transition metals compared to 4d transition metals.^56,57^ In conjunction with this slow substitution rate, the use of alternative axial ligands, like carboxylates, on the Os245 scaffold could result in new analogues with very long biological activation times.

**Chart 2:**
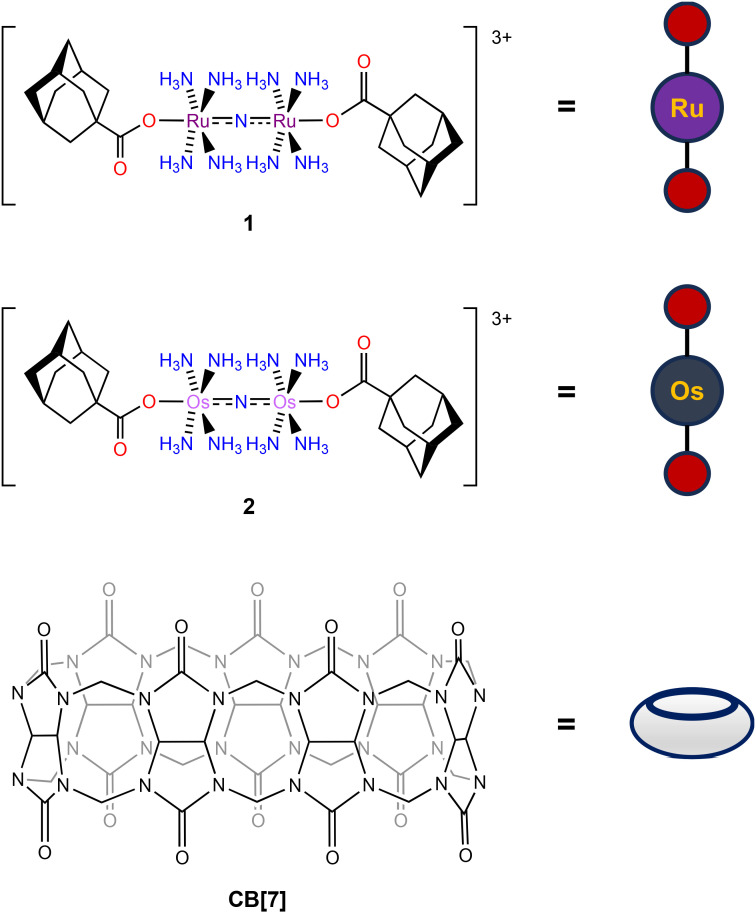
Structures of 1, 2, and CB[7] with their pictorial representations used within this manuscript.

Although we have successfully shown in earlier studies that modification of the axial carboxylates of Ru265 analogues could sufficiently alter their properties, we sought alternative approaches to finetune their behavior in biological solutions. In this regard, the use of supramolecular complexes and interactions has recently been demonstrated to have promising biological applications. For example, biologically active guest molecules can exhibit substantially different solubilities, cell permeabilities, and pharmacokinetic properties once encapsulated in a supramolecular host.^58–60^ The most widely used host molecules for these applications are cyclodextrins and cucurbiturils. In particular, cucurbit-[*n*]-uril (CB[*n*]) has been shown to be a highly effective host for a number of different molecular architectures and has had substantial impacts on their biological properties.^58–63^ A significant example demonstrated that the encapsulation of pentylenetetrazol, a GABA-A receptor antagonist, within CB[7] was able to attenuate its dose-limiting side of effect of seizure induction *in vivo*.^64^ Given that the *in vivo* applications of both Ru265 and Os245 are also dose-limited by seizure induction,^50,52^ we hypothesized that the encapsulation of these MCU inhibitors within CB[7] would be a promising approach to modify and potentially improve their biological properties. To make these compounds amenable for CB[7] encapsulation, we appended them with axial carboxylate adamantane ligands. Adamantane, a well-known lipophilic moiety in drug development,^65^ is a high affinity guest molecule for CB[7], making the resulting host–guest complex valuable for biological applications.^66–78^ In prior studies, the covalent attachment of this group to a cisplatin derivative enabled its encapsulation in CB[7], validating this approach for metal-based compounds.^79^ Thus, we sought to perform a similar functionalization of Ru265 and Os245 for their encapsulation into CB[7]. In this work, we report the synthesis and characterization of adamantane-functionalized derivatives of Ru265 and Os245 (1 and 2, Chart 2), an examination of their supramolecular complex formation with CB[7], and *in vitro* and *in vivo* investigations of the biological ramifications of this complex formation. Collectively, these studies reveal that the addition of CB[7] to these complexes increase their cellular accumulation without sacrificing their MCU-inhibitory activities.

## Results and discussion

### Synthesis and characterization

Compounds 1 and 2 were designed to be guest molecules for CB[7] by incorporation of 1-adamantanecarboxylate (OAd^−^) ligands on the axial positions. The adamantane group provides a high-affinity guest for CB[7], and the carboxylate has been established by us to be a suitable axial ligand for this compound class. The syntheses of these complexes commenced from Ru265′ ^51,53^ and Os245′.^50^ The ligand 1-adamantanecarboxylic acid was converted to the sodium 1-adamantanecarboxylate (NaOAd) salt *via* the reaction with one equiv. sodium hydroxide in ethanol. To access 1, a procedure that we had previously developed for related carboxylate-functionalized analogues was applied.^53–55^ Specifically, Ru265′ was treated with two equiv. of NaOAd in MeOH, while heating the mixture at 60 °C for 16 h. Subsequent crystallization of the crude product from this reaction *via* vapor diffusion of hexane into THF afforded analytically pure 1. Compound 2 was synthesized under similar conditions by treating Os245′ with two equiv. NaOAd in MeOH at 60 °C. Given the greater inertness of Os, however, the 48 h reaction time required for formation of 2 was substantially longer than that needed for 1. Upon purification of the crude product by low-temperature evaporation of THF, analytically pure 2 was obtained as a yellow crystalline solid. Importantly, 2 is the first example of an Os245 analogue bearing axial carboxylate ligands, and this synthetic procedure should therefore be broadly applicable for making new derivatives of this MCU inhibitor. CB[7] used within these studies was synthesized according to previous literature procedures.^80,81^

**Fig. 1:**
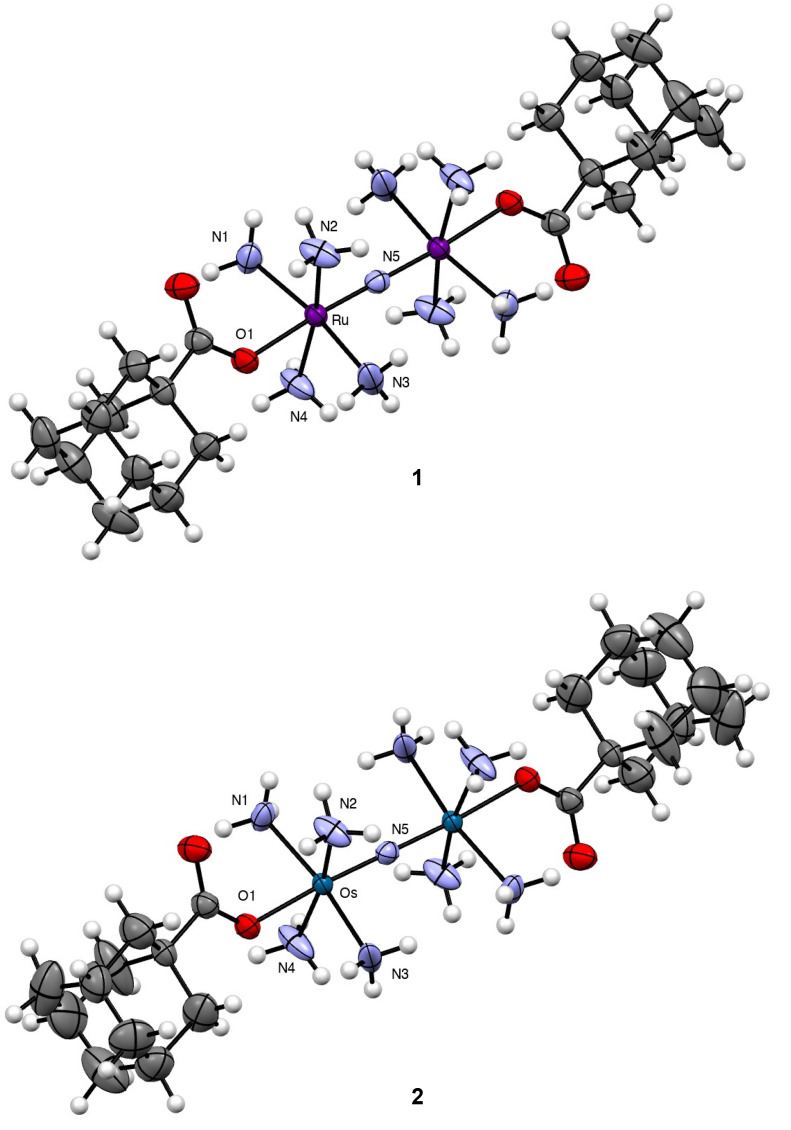
Crystal structures of 1 and 2. Outer-sphere counterions and solvent molecules are omitted for clarity. Thermal ellipsoids are shown at the 50% probability level. Crystallographic information for both complexes is given in Table S1.† Key interatomic distances and angles are given in Table 1. All interatomic distances and angles are given in Table S2.†

Both compounds, as well as NaOAd and CB[7], were characterized *via* IR and NMR (Fig. S1–S14†) spectroscopies, elemental analysis, and X-ray crystallography (Fig. 1 and Tables S1–S2†). The ^1^H NMR spectra of 1 and 2 in DMSO-*d*_6_ showed diagnostic resonances arising from the OAd^−^ ligand. In particular, the hydrogen atoms of this ligand appear at 1.92, 1.71, and 1.63 ppm for both 1 and 2. Consistent with coordination to the metal centers, these chemical shifts are slightly downfield of the resonances for free NaOAd salt, which appear at 1.87, 1.70, and 1.60 ppm. In addition, the ^1^H NMR spectra also reveal broad singlets at 3.90 and 4.74 ppm for 1 and 2, which can be assigned to the protons of the coordinated NH_3_ ligands. For 1, the resonances are slightly shifted upfield by 0.2 ppm compared to the parent compound Ru265, but are comparable to other carboxylate-capped analogues whose resonances span the range of 3.90–3.95 ppm.^53–55^ For 2, the resonance of these NH_3_ hydrogen protons is similarly shifted 0.2 ppm upfield, comparable to that of Os245′. Because 2 is the first example of carboxylate-capped Os analogue of this compound class, this resonance can provide a diagnostic means of characterization. Within the IR spectra of 1 and 2, the asymmetric M–N–M stretching modes are tentatively assigned to be 1049 and 1116 cm^−1^, respectively. These energies are comparable to the stretching modes found in the parent Ru265 and Os245 complexes at 1050 and 1108 cm^−1^, indicating that axial ligand substitution has little effect on the metal-nitrido bond. The higher energy of the M–N–M stretching mode of 2 compared to 1 suggests stronger π-bonding in the Os–N–Os moiety than that of the Ru–N–Ru.

Single crystals of both complexes were analyzed by X-ray diffraction. The crystal structures of their Ru- and Os-containing cations are shown in Fig. 1 with relevant interatomic distances and angles shown in Table 1 and Table S2.† Both complexes form isomorphous crystals, attaining the same space group and nearly identical unit cell parameters. Consequently, their structural features are largely similar. The bridging N^3−^ of both complexes resides on a crystallographic inversion center. As such, the M–N–M angle is perfectly linear for 1 and 2, and the four NH_3_ ligands on each metal center are arranged in an eclipsed conformation. The OAd^−^ ligands occupy the position *trans* to the N^3−^ and are supported by additional intramolecular hydrogen bonds with the equatorial NH_3_ ligands. Within 1, the Ru–N and Ru–O interatomic distances are similar to those for previously reported carboxylate-functionalized derivatives of Ru265.^53–55^ As noted previously, the strong *trans* influence of the N^3−^ bridging ligand leads to elongation of the axial Ru–O distances compared to mononuclear analogues. The interatomic distances found within 2 are nearly the same as those of 1, a property that reflects the similar ionic radii of these metals.

**Table 1:** Key interatomic distances and angles for 1 and 2 a

	1	2
M–N(5) (Å)	1.7424(3)	1.7685(3)
M–O(1) (Å)	2.093(3)	2.105(5)
M–N(5)–M (°)	180.0	180.0
N(5)–M–O(1) (°)	176.44(9)	176.52(14)

a Atoms are labelled as shown in Fig. 1.

### Encapsulation into CB[7]

**Fig. 2:**
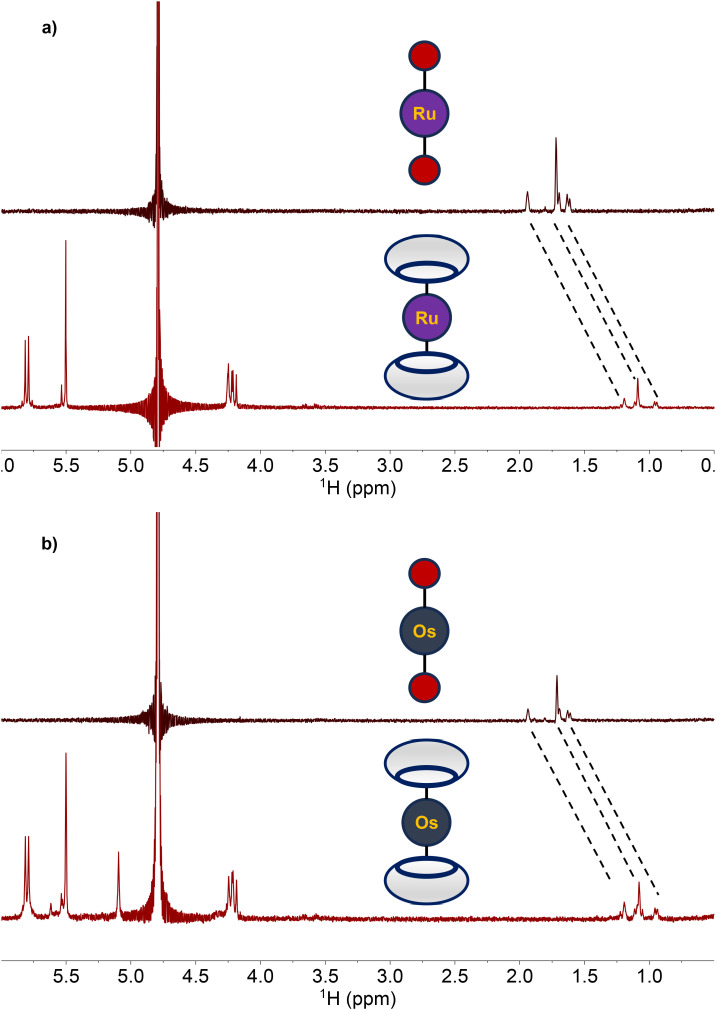
(a) ^1^H NMR (500 MHz, 25 °C) spectra in D_2_O of 1 (top) and 1 + two equiv. CB[7] (bottom). (b) ^1^H NMR (500 MHz, 25 °C) spectra in D_2_O of 2 (top) and 2 + two equiv. CB[7] (bottom). The shift of the adamantane ^1^H resonances are indicated with the dotted lines.

With the adamantane-functionalized 1 and 2 fully characterized, their capacity to act as guests for the supramolecular host molecule CB[7] was investigated. Upon the addition of two equiv. CB[7] to either 1 and 2 in D_2_O, substantial changes in the ^1^H NMR resonances of these complexes were observed. Specifically, resonances of the adamantane functional group in both 1 and 2 shifted upfield by nearly 0.6 ppm (Fig. 2). This upfield shift is a consequence of the encapsulation of adamantane within these hosts, as observed previously.^79,82,83^

Encapsulation of 1 and 2 within CB[7] was further confirmed through 2D diffusion-ordered NMR experiments (Fig. S15 and S16†). Using this method, we measured the diffusion constants (*D*) of the free hosts and guests, as well as the putative host–guest complex, by implementing a pulse-gradient spin echo experiment (PGSE) in conjunction with the mathematical relationship outlined in eqn (1) and (2),^84^

1 ln(*I*/*I*_0_) = −*DG*^2^

2 *G*^2^ = *γ*^2^*δ*^2^*g*^2^(*Δ* − (*δ*/3))

where *I*/*I*_0_ is the normalized intensity of each signal after the applied gradient pulse (*G*^2^), which is a mathematical function of the gyromagnetic ratio (*γ*), the duration (*δ*) and separation (*Δ*) of the gradient pulses, and the gradient pulse amplitude (*g*). The data obtained from this experiment were analyzed with a Stejskal-Tanner plot, the linear slope of which yields a numerical value for *D* (Table 2 and Fig. S17†).^84^ Under these conditions, the *D* value for CB[7] was determined to be 3.14 × 10^−6^ cm^2^ s^−1^. This value is consistent with those reported in the literature, which range from 3.07–3.23 × 10^−6^ cm^2^ s^−1^,^83,85^ providing validation of our experimental methodology. Both 1 and 2, have a similar *D*, consistent with their identical structures and sizes. Upon addition of two equiv. of CB[7] to these complexes, a notable decrease in *D* is observed. This change is consistent with expected increase in hydrodynamic radius upon supramolecular complex formation, as has been observed for other systems in the literature.^85^ Further, all ^1^H resonances on both the host and guest molecules have the same *D*, indicating the formation of a supramolecular complex.

**Table 2:** Diffusion constant (*D*) of each species calculated by PGSE (Fig. S17†)^a^

Compound	*D* (× 10^−6^ cm^2^ s^−1^)
CB[7]	3.14 ± 0.09
1	3.40 ± 0.14
1 + CB[7]	2.10 ± 0.04
2	3.11 ± 0.18
2 + CB[7]	2.18 ± 0.05

a Determined at 25 °C in D_2_O at 1 mM for 1 and 2 and 2 mM for CB[7].

### Binding constant determination

To quantify the strengths of the interactions of 1 and 2 with CB[7], isothermal titration calorimetry (ITC) was used to measure the binding constants (*K*_a_) and thermodynamic properties of each complex with CB[7] (Fig. S18–S20†). ITC has been widely used in biophysical chemistry to study the interactions between biomolecules, but is also broadly applicable to other systems like supramolecular host–guest complexes.^86^ The binding analysis of 1 and 2 with CB[7] by this method was carried out using 50 mM MOPS as a buffer, which has an affinity for CB[7].^87^ Accordingly, the data analysis and fitting to acquire thermodynamic parameters were adjusted to account for this competitive equilibrium.^88^ The ITC thermograms (Fig. S18–S20†) reveal steep binding isotherms that are characteristic of large *K*_a_ values. Fitting of these thermograms, accounting for competitive MOPS binding, was used to obtain the *K*_a_ values and binding stoichiometries (*n*), shown in Table 3. The free ligand NaOAd interacts with CB[7] *via* the formation of a 1 : 1 complex and a binding constant of 8 × 10^6^ M^−1^. Consistent with the presence of two adamantyl guest moieties on both 1 and 2, their binding stoichiometries were determined to be 2 : 1 (*n* ≈ 0.5), a property that was also confirmed by NMR spectroscopy (Fig. S21–S23†). Additionally, the encapsulation of these complexes were not affected by the presence of biologically relevant metal ions Mg^2+^, Ca^2+^, and Zn^2+^, as evidenced by NMR spectroscopy (Fig. S24†). We also note that some biologically relevant organic molecules, such as cholesterol, are guests of CB[7]. The large *K*_a_ values of our complexes, conferred by the adamantane functional group, should limit their displacement under biological conditions. Notably, the *K*_a_ values for the interaction of 1 and 2 with CB[7] were similar and roughly two orders of magnitude larger than that of free NaOAd. In addition, only a single *K*_a_ value was measured for both complexes, indicating that the addition of the second CB[7] is most likely not cooperative and occurs concurrently with the first. The *K*_a_ values for CB[7] interacting with 1 and 2 are two orders of magnitude smaller compared to the cisplatin-aminoadamantane complex mentioned previously, which has a *K*_a_ with CB[7] of 7.26 × 10^10^ M^−1^.^79^ Aminoadamantane itself has a substantially higher affinity for CB[7] (*K*_a_ = 1.7 × 10^14^ M^−1^)^89^ than NaOAd, most likely due to the additional hydrogen-bonding NH_2_ groups that are available on this compound to stabilize the host–guest complex. In any case, the 10^8^ M^−1^ of these *K*_a_ values are still sufficiently large for biological applications.

**Table 3:** Thermodynamic binding data of NaOAd, 1, and 2 measured by ITC in pH 7.4 MOPS-buffered solution at 25 °C^a^

Compound	Δ*H*_ITC_ (kJ mol^−1^)	*n*	*K*_a_ (M^−1^)
NaOAd	−6.3 ± 0.4	1.01 ± 0.01	(8.7 ± 0.8) × 10^6^
1	−31 ± 2	0.6 ± 0.1	(4 ± 2) × 10^8^
2	−32 ± 1	0.7 ± 0.1	(6 ± 2) × 10^8^

a Values are obtained from data fits (Fig. S18–S20†) and a *post hoc* analysis for *K*_a_ accounts for the competitive binding of the MOPS buffer.

### Aquation kinetics

Carboxylate-functionalized analogues of Ru265 undergo aquation with half-lives ranging from 2–10 h. Accordingly, the aquation kinetics of 1 and 2 were determined by UV-vis spectroscopy in pH 7.4 MOPS buffer at 37 °C (Fig. S25 and S26†). The aquation half-lives of 1 and 2 were determined by monitoring the decrease in the peaks at 258 nm and 244 nm, respectively, over time. As noted previously, UV-vis spectroscopy cannot suitably resolve the individual aquation steps of both the first and second axial ligands for this compounds class.^54,55,90^ However, an exponential fit of these data, corresponding to a simplified first-order process, is able to accurately capture the pseudo-first order rate constants (*k*_obs_) for the aquation of the first carboxylate, which we have previously found to be substantially smaller than that of the second.^53^ An exponential fit of the UV-vis spectra of 1 and 2 revealed aquation half-lives (*t*_1/2_) of 7.5 and 29.9 h (Table 4). The 7.5 h half-life of 1 is within the range previously observed for other carboxylate-capped Ru compounds of this class.^53–55^ The slower aquation kinetics for 2 are expected based on the greater inertness of 5d transition metals. The extended half-life of 2 suggests that it may stay intact longer in biological settings compared to 1. To assess the impact of encapsulation within CB[7], the aquation kinetics of 1 and 2 were measured in the presence of two equiv. of this host molecule. As shown in Table 4, encapsulation of 1 and 2 had no significant effect on the rates of aquation of these compounds. This result is consistent with a comparatively rapid on–off preequilibrium of encapsulation, followed by the slow rate-determining aquation of the first carboxylate ligands.

**Table 4:** Aquation kinetic data^a^ of 1 (25 μM) and 2 (25 μM) in the presence or absence of CB[7] (50 μM)

Compound	*k*_obs_ (× 10^−5^ s^−1^)	*t*_1/2_ (h)
1	2.77 ± 0.33	7.96 ± 0.85
1 + CB[7]	2.48 ± 0.31	7.85 ± 0.98
2	0.645 ± 0.33	29.91 ± 1.56
2 + CB[7]	0.676 ± 0.41	28.48 ± 1.69

a Data were collected using UV-vis spectroscopy in 100 mM MOPS-buffered solution (pH 7.4) at 37 °C. All data are presented as the average of three independent trials ± SD.

### Cytotoxicity and cellular uptake

Having characterized 1 and 2 and studied their supramolecular encapsulation properties, their *in vitro* biological activities were probed. To effectively use these compounds as tools or therapeutic agents related to _*m*_Ca^2+^ overload, they need to ideally be non-cytotoxic in human cells. Thus, their cytotoxicity was assessed in HeLa cells using the colorimetric thiazolyl blue tetrazolium bromide (MTT) assay (Fig. S27†). After a 72 h incubation period, 2 was effectively non-toxic even at its highest administered concentration of 100 μM. By contrast, 1 was moderately cytotoxic under these conditions with a 50% growth inhibitory concentration (IC_50_) of 21.9 ± 2.0 μM. The addition of two equiv. of CB[7] did not change the lack of cytotoxicity of 2; however, two equiv. of CB[7] did attenuate the cytotoxicity of 1. As a further control experiment, the cytotoxicity of the ligand, NaOAd in the presence and absence of CB[7] was determined, revealing it to be non-toxic under any of these conditions. The moderate cytotoxicity of 1 is therefore enigmatic, given that both Ru265′ and NaOAd are non-toxic, but is unlikely to have a significant impact for downstream applications of this compound as a nM inhibitor of _*m*_Ca^2+^ uptake. In addition, the JC-1 assay was employed to verify that compounds 1 and 2 do not negatively affect the mitochondrial membrane potential of HeLa cells treated with 50 μM for 24 h. The presence of two equiv. of CB[7] with these complexes also did not negatively affect mitochondrial function (Fig. S28 and S29†).

**Fig. 3:**
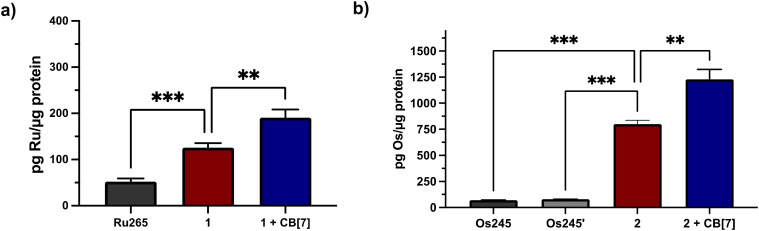
Cellular accumulation of (a) Ru (measured by GFAAS) and (b) Os (measured by ICP-MS) in the lysates of 10^5^ HeLa cells that were dosed with 50 μM of each complex ± 100 μM CB[7] for 24 h. Values were normalized to the total protein content within the cell lysates as determined by the BCA assay. Results are reported as the average of three independent biological replicates ± SD. **p < 0.01, ***p < 0.001; *n* = 3.

Given the need for cell-permeable MCU inhibitors, the cell uptake in HeLa cells of complexes 1 and 2 in the presence and absence of two equiv. CB[7] was investigated. The HeLa cells were treated with 50 μM of either 1 and 2 with or without 100 μM CB[7] for 3 h. The cells were then washed, collected, and lysed, and the protein content was measured *via* the bicinchoninic acid (BCA) assay. For 1, the Ru content was determined *via* graphite furnace atomic absorption spectroscopy (GFAAS), whereas for 2, the Os content was determined *via* inductively-coupled plasma mass spectrometry (ICP-MS). The cell uptake of these complexes is reported as the total metal concentrations measured normalized to the protein content of the cells, and these data are shown in Fig. 3. Both 1 and 2 are taken up at higher levels under these conditions compared to Ru265 and Os245, consistent with the presence of the lipophilic adamantane group. In the presence of two equiv. of CB[7], the uptakes of 1 and 2 increase further, suggesting that the CB[7] facilitates their transport through the cell membrane. Additionally, in both the presence and absence of CB[7], 1 is significantly accumulated within the mitochondria of HeLa cells dosed with the complex (Fig. S30†). As the cellular uptake properties of 1 and 2 are similar, we did not measure the subcellular localization of 2, but expect it to also accumulate within the mitochondria. These similar uptake properties bring into question the mechanism of cellular uptake of these complexes with and without CB[7]. We have previously reported that Ru265′ and Os245′ are readily taken up into cells through the organic cation transporter 3 (OCT3).^50,51^ Our studies on other carboxylate derivatives have suggested an alternative mechanism.^53^ To probe mechanistic interactions for these complexes, we co-incubated 1 ± two equiv. CB[7] with a variety of uptake mechanism inhibitors – specifically an inhibitor of OCT3, an inhibitor of an energy-dependent uptake pathway, and CuSO_4_ to saturate the CTR1 transporter (Fig. S31†). In all scenarios, the cellular accumulation was not significantly reduced, indicating that further mechanistic studies need to be probed to determine the cellular uptake pathway of these complexes. Given the favorable properties associated with the CB[7] supramolecular complexes, this host molecule was assessed in more detail in subsequent investigations described below.

### _*m*_Ca^2+^ uptake inhibition

**Fig. 4:**
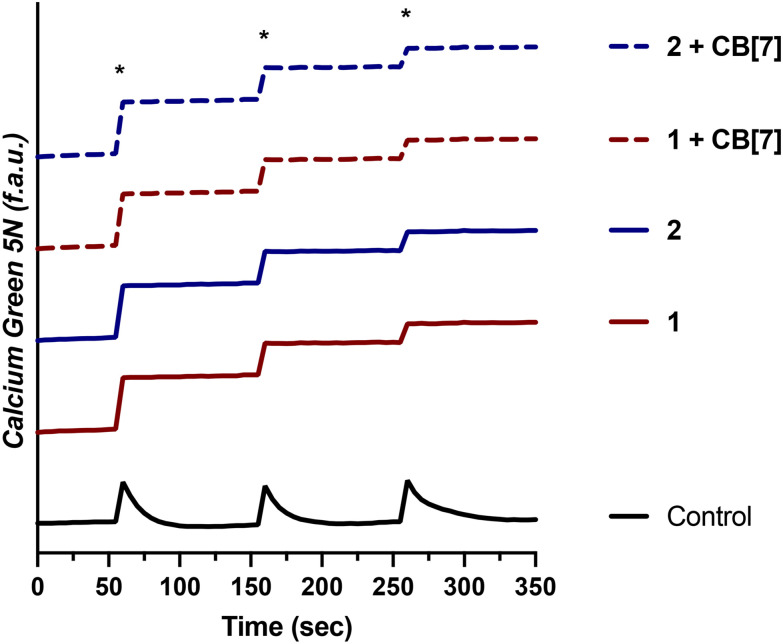
Cytosolic Ca^2+^ transients in the presence or absence of 25 μM 1 and 2 ± two equiv. CB[7] measured with the cytosolic Ca^2+^ dye Calcium Green 5 N in permeabilized HEK293T cells (1 × 10^7^ cells per mL). CaCl_2_ (20 μM) boluses are indicated with an asterisk (*).

Having demonstrated that 1 and 2, as well as their supramolecular complexes with CB[7], exhibit good cell uptake and minimal cytotoxicity, we next sought to examine the MCU-inhibitory activity of these complexes in both permeabilized and intact cells. In digitonin-permeabilized HEK293T cells, the parent complexes Ru265, Os245, and Os245′ all show potent, nanomolar _*m*_Ca^2+^ uptake inhibition, and within intact cells they can abrogate _*m*_Ca^2+^ uptake completely when administered at a concentration of 50 μM.^47,50^ To assess the _*m*_Ca^2+^ uptake of the new analogues 1 and 2, as well as their supramolecular complexes with CB[7], they were first tested at a single-dose concentration of 25 μM in the presence or absence of 50 μM CB[7] in permeabilized HEK293T cells. As shown in Fig. 4, both 1 and 2 completely inhibit _*m*_Ca^2+^ uptake with and without CB[7] at this concentration. Notably, the free ligand (NaOAd) showed no inhibitory activity of _*m*_Ca^2+^ uptake up to 50 μM (Fig. S32†). Building upon this initial result, a dose–response analysis of inhibitory activity was carried out to determine the relative potencies of 1, 2, and their supramolecular CB[7] complexes, in comparison to Ru265 and Os245′ (Fig. S33†). As shown in Table 5, both 1 and 2, when administered immediately, inhibit _*m*_Ca^2+^ with nanomolar efficacies, but are significantly less potent than Ru265 and Os245′. In addition, the presence of CB[7] does not significantly affect the potency of these complexes in this permeabilized cell model, suggesting that the complexes can readily dissociate from this host and interact with the putative MCU target. The release of supramolecular materials from metal-based complexes has previously been established,^91^ confirming that this property is common in these constructs. The IC_50_ value of 1 is comparable to those of other carboxylate-derivatives of Ru265 reported, at least when comparing their activities prior to aquation.^54,55^ Complex 2 is the first carboxylate-capped analogue of Os245, and therefore its activity cannot be compared to related derivatives. In comparison to the aquated Os245′, however, compound 2 is approximately 40× less potent. By contrast, the difference in potencies between the Ru analogues is less substantial; compound 1 is only 5× less effective than Ru265. The larger inhibitory activity differential for the Os complexes is attributed to the slower aquation kinetics of 2 in comparison to 1. As the aquation data discussed above show, the aquation of Os245 occurs with a half-life of 12 h,^50^ whereas the half-life for 2 is 30 h, 2.5 times longer. In comparing the IC_50_ values, it is also apparent that that of 2 is 2.5 times larger than Os245. Thus, the inhibition of _*m*_Ca^2+^ uptake primarily mediated by the aquated form Os245′.

**Table 5:** IC_50_ of _*m*_Ca^2+^ uptake inhibition in permeabilized HEK293T cells (1 × 10^7^ cells per mL). Data presented as the average of three independent trials ± SD

Compound	IC_50_ (nM)
Ru265^a^	8.6 ± 2.2
1	50.0 ± 0.8
1 + CB[7]	41.3 ± 3.1
Os245	90.9 ± 1.3
Os245′	5.7 ± 0.3
2	249.4 ± 5.6
2 + CB[7]	261.0 ± 4.3

a Ref. 48.

**Fig. 5:**
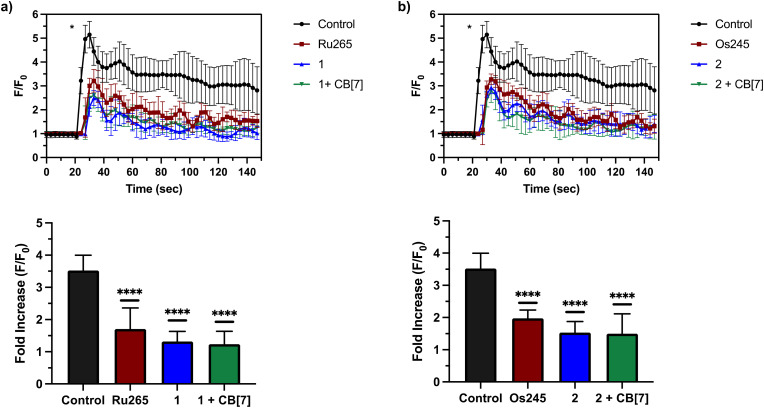
(a) _*m*_Ca^2+^ transients in HeLa cells after treatment with histamine (100 μM), indicated with an asterisk (*), that were pretreated with or without Ru265 (50 μM), 1 (50 μM), and 1 (50 μM) + CB[7] (100 μM). Fold increase (*F*/*F*_0_) of the fluorescence response of each treatment upon addition of histamine (100 μM) are also shown. The maximum *F*/*F*_0_ is presented for each experiment. (b) _*m*_Ca^2+^ transients in HeLa cells after treatment with histamine (100 μM), indicated with an asterisk (*), that were pretreated with or without Os245 (50 μM), 2 (50 μM), and 2 (50 μM) + CB[7] (100 μM). Fold increase (*F*/*F*_0_) of the fluorescence response of each treatment upon addition of histamine (100 μM) are also shown. The maximum *F*/*F*_0_ is presented for each experiment. Data are presented as the mean response ± SD. ****p < 0.001; ns = not significant; *n* = 3.

**Fig. 6:**
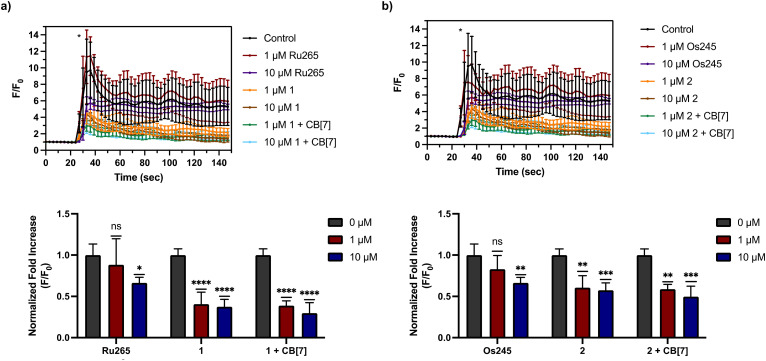
(a) _*m*_Ca^2+^ transients in HeLa cells after treatment with histamine (100 μM), indicated with an asterisk (*), that were pretreated with or without Ru265 (1 or 10 μM), 1 (1 or 10 μM), and 1 (1 or 10 μM) + CB[7] (2 or 20 μM). Fold increase (*F*/*F*_0_) of the fluorescence response of each treatment upon addition of histamine (100 μM) are also shown. The maximum *F*/*F*_0_ is presented for each experiment. (b) _*m*_Ca^2+^ transients in HeLa cells after treatment with histamine (100 μM), indicated with an asterisk (*), that were pretreated with or without Os245 (1 or 10 μM), 2 (1 or 10 μM), and 2 (1 or 10 μM) + CB[7] (2 or 20 μM). Fold increase (*F*/*F*_0_) of the fluorescence response of each treatment upon addition of histamine (100 μM) are also shown. The maximum *F*/*F*_0_ is presented for each experiment. Data are presented as the mean response ± SD. *p < 0.1, **p < 0.01, ***p < 0.001, ****p < 0.0001; ns = not significant; *n* = 3.

After assessing their permeabilized cell inhibitory activities, 1, 2, and their CB[7] complexes were evaluated in intact cell systems. In these studies, HeLa cells were incubated with the Ca^2+^-responsive mitochondria-localizing fluorescent sensor Rhod2-AM, stimulated with histamine to trigger _*m*_Ca^2+^ uptake, and then imaged by confocal fluorescence microscopy to measure _*m*_Ca^2+^ levels. When these HeLa cells were treated with 50 μM of 1 or 2, a significant decrease of _*m*_Ca^2+^ uptake was detected after stimulation with histamine (Fig. 5). Furthermore, the presence of two equiv. of CB[7] did not negatively impact the inhibitory properties of these compounds under these conditions. After demonstrating 50 μM to be an effective concentration, we investigated the abilities of these complexes to operate at lower concentrations. As shown in Fig. 6, both 1 and 2, in the presence or absence of CB[7], were able to inhibit _*m*_Ca^2+^ uptake, whereas the parent complexes Ru265 and Os245 were not effective at these lower concentrations. This result is consistent with the cellular uptake studies that show 1 and 2 to be taken up by cells at much higher concentrations than Ru265 and Os245.

### Protection against oxygen glucose deprivation

To determine the functional significance of the _*m*_Ca^2+^ uptake inhibitory properties of 1 and 2, we investigated them in an *in vitro* model of ischemic stroke, oxygen glucose deprivation (OGD).^50,52^ Because _*m*_Ca^2+^ overload is a key factor that leads to neuronal cell death in ischemic stroke,^92^ we sought to assess the cytoprotective effects of 1, 2, and their CB[7] complexes. Previously, we have demonstrated that Ru265 and Os245 protect cortical neurons against an OGD model, a consequence of their ability to prevent _*m*_Ca^2+^ overload. As shown in Fig. 6, lower concentrations of 1 and 2 in the presence or absence of CB[7] are more effective _*m*_Ca^2+^ uptake inhibitors in intact cells than Ru265 or Os245, suggesting that they might also exhibit better cytoprotective effects against this *in vitro* OGD model.

**Fig. 7:**
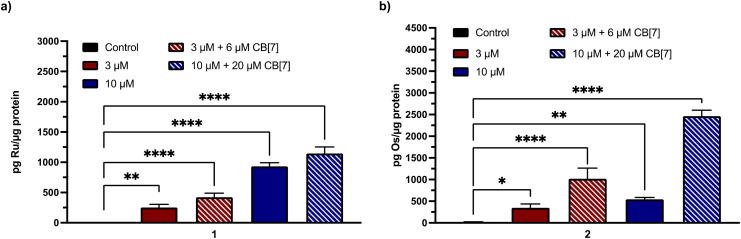
Cellular accumulation of (a) Ru (measured by GFAAS) and (b) Os (measured by ICP-MS) in primary cortical neuron cell lysates dosed with 3 or 10 μM (a) 1 or (b) 2 ± 6 or 20 μM CB[7] for 24 h. Values were normalized to the total protein content within the cell lysates as determined by the BCA assay. Results are reported as the average of three independent biological replicates ± SD. *p < 0.1, **p < 0.01, ****p < 0.0001; ns = not significant; *n* = 3.

To validate that the cell uptake properties of these complexes remain consistent in primary cortical neurons, we performed GFAAS and ICP-MS studies on cells treated with 3 or 10 μM 1 or 2 in the presence or absence of CB[7] for 24 h. After normalization to the protein content by the BCA assay, the data in Fig. 7 show that both 1 and 2 are taken up effectively by these cortical neurons. Significantly, the addition of two equiv. of CB[7] further increased the neuronal uptake of these compounds, consistent with the cellular uptake data measured within HeLa cells (Fig. 3).

**Fig. 8:**
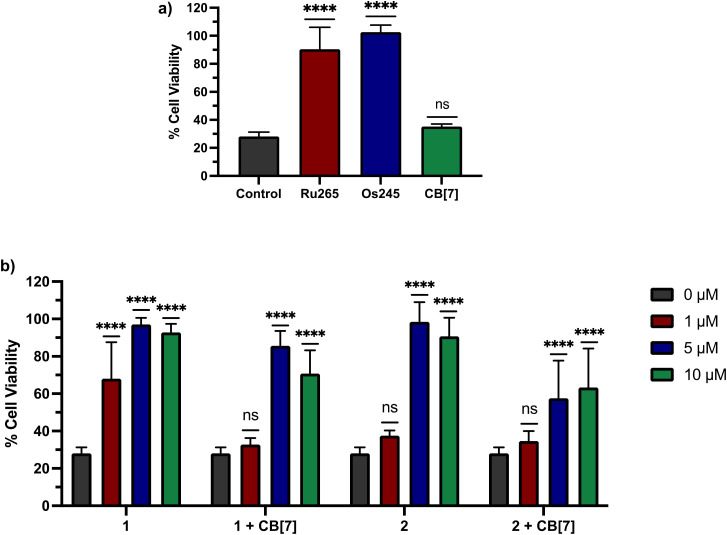
Cell viability in primary cortical neuron cultures subjected to a lethal period of OGD (90 min) in the presence of (a) Ru265 (10 μM), Os245 (50 μM), or CB[7] (20 μM) and (b) increasing concentrations of 1 or 2 in the presence and absence of two equiv. CB[7] for 3 h. Cell viability was measured using the MTT assay 24 h after OGD. Bars represent the mean ± SD. ****p < 0.0001; ns = not significant.

Based on these cell uptake data, we proceeded to analyze the protective effects of these compounds against OGD in primary cortical neurons. Consistent with previous results, Ru265 (10 μM) and Os245 (50 μM) preserve the cell viability of cortical neurons subjected to OGD (Fig. 8a). By contrast, CB[7] (20 μM) showed no protective effects (Fig. 8a). We next investigated 1 and 2 in the presence or absence of two equiv. CB[7] within this model. Both compounds 1 and 2 show protective effects against OGD, even at concentrations as low as 1 and 5 μM, respectively (Fig. 8b). The protective effects of 1 at this concentration are consistent with its ability to inhibit _*m*_Ca^2+^ uptake in intact cells at this concentration as well (Fig. 6). By contrast, although 2 is also able to inhibit _*m*_Ca^2+^ uptake in intact cells at 1 μM (Fig. 6), it fails to elicit any protection under these conditions. The poorer cytoprotection conferred by 2 is consistent with the similarly poorer protective effects of Os245 compared to Ru265.^50^ Thus, this trend appears to be a recurring phenomenon in comparing Ru and Os analogues of these inhibitors, and it may be a consequence of their different ligand substitution kinetics. The addition of two equiv. CB[7] to 1 and 2 altered their protective effects. At 1 μM, the protective effects observed by 1 are completely eliminated in the presence of two equiv. CB[7], indicating that supramolecular encapsulation can have an impact on its biological properties. However, at higher concentrations, the addition of CB[7] did not affect the cytoprotective properties of 1 and 2. This result is somewhat unexpected because we demonstrated that CB[7] can increase the cell uptake of these complexes. A possible explanation is that the intracellular localization of the CB[7]-encapsulated compounds is unfavorable for _*m*_Ca^2+^ uptake inhibition.

### *In vivo* biological properties

**Fig. 9:**
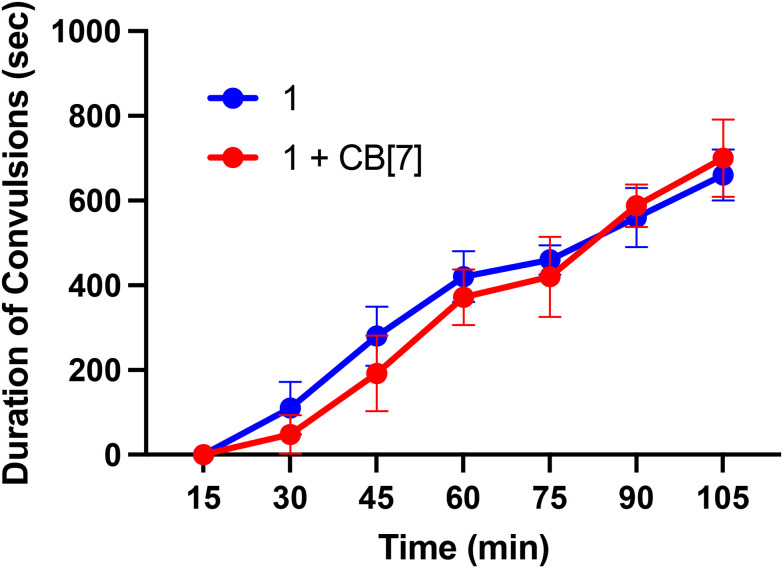
Seizure duration in male C57/BI6 mice injected i.p. with 10 mg kg^−1^ of 1 or 1 + two equiv. CB[7].

Finally, we sought to investigate *in vivo* properties of these complexes in the presence and absence of CB[7]. As noted above, when administered to mice at doses greater than 10 mg kg^−1^, Ru265 and Os245 cause seizures.^50,52^ This side effect significantly attenuates the therapeutic windows of these compounds. The mechanism by which these compounds induce convulsions is currently unknown, but this phenomenon has also been observed with RuRed.^50,52,93–95^ Furthermore, a recent study has suggested that the off-target inhibition P/Q-type^96^ Ca^2+^ and KCNQ^97^ K^+^ channels by Ru265 may be an important contributing factor to this seizure activity.^98^ In this context, a related study, mentioned previously, showed that CB[7] can attenuate the seizure-induction of pentylenetetrazol in mice, thus prompting us to investigate the ability of this host molecule to modulate the *in vivo* properties of 1. Male C57/BI6 mice were intraperitoneally (i.p.) injected with 1 at a dose of 10 mg kg^−1^ in the absence and presence of 20 mg kg^−1^ CB[7], which coincides with a 1 : 2 molar stoichiometry of 1 to CB[7]. These mice were then monitored for seizure-like behaviors, including whisker trembling, motionless staring, facial jerking, and clonic convulsions. As shown in Fig. 9, the duration of the convulsions increased with time after the injection of 1, showing the enhanced severity of the side effects. Notably, the presence of CB[7] failed to elicit any change in this time course of events, showing that it failed to protect mice against the seizure induction of 1. Thus, although we demonstrated that *in vitro* CB[7] can modulate the biological properties, these effects do not translate *in vivo*. This result may be a consequence of faster ligand substitution of the guest adamantyl carboxylate ligands of 1*in vivo* compared to *in vitro*. Based on these results, further studies involving 2 were not undertaken in order to minimize animal usage and distress. Because of its structural similarity to 1, as well as the seizure-inducing side effects of both Os245 and Ru265,^50,52^ we do not expect CB[7] to improve the *in vivo* tolerability of 2. Installing a guest ligand on the non-leaving ammine ligands of 1 may be a better strategy for enabling the benefits of this host–guest chemistry *in vivo*, as it is likely that the parent Ru265′ or Os245′ compounds are directly responsible for the seizure-inducing behavior *in vivo*.

## Conclusions

In this study, we reported the synthesis and characterization of two new derivatives of Ru265 and Os245 with adamantane carboxylate groups appended to the axial site. Compound 1 expands upon the list of previously reported carboxylate-functionalized analogues of Ru265,^53–55^ and compound 2 is the first reported carboxylate-functionalized analogue of Os245. Both 1 and 2 interact strongly with CB[7], forming 2 : 1 complexes. When investigated in biological models, the role of the CB[7] complexation is somewhat enigmatic. CB[7] enhances the cellular uptake of both compounds but does not appear to influence their permeabilized or intact cell _*m*_Ca^2+^ uptake-inhibitory properties. Within the *in vitro* OGD model, only the protective effects of 1 at low concentrations are affected by CB[7]. Lastly, CB[7] fails to attenuate *in vivo* seizure activity of 1 in mice. The poor success of this CB[7] encapsulation approach therefore suggests that alternatives are needed. It is well-documented in other systems that CB[7] can improve cell uptake, decrease toxic side effects, and enhance biological activities of promising drug candidates that are strongly encapsulated by it.^58–63^ A possible reason for the poor performance of the CB[7] complexes of 1 and 2 in the biological models may be a consequence of the fact that the guest adamantyl moiety is attached to the carboxylate leaving group ligands of these complexes. As such, the effects of the CB[7] encapsulation may be negated *via* departure of these axial ligands. This hypothesis suggests that an alternative approach involving the attachment of adamantyl groups to the amine ligands that remain bound to the metal centers may be more fruitful. In any case, a remarkable discovery within this study is that the adamantyl group of 1 and 2 provide substantial advantages over the parent complexes Ru265 and Os245. Most notably, 1 and 2 are effective _*m*_Ca^2+^ uptake inhibitors and cytoprotective agents at concentrations lower than those needed for Ru265 and Os245. These results highlight that optimization of the pharmacokinetic properties of these compounds *via* axial ligand modification provides a promising strategy for accessing more effective and biologically useful inhibitors.

## Data availability

The data supporting this article have been included as part of the ESI.† Crystallographic data for 1 and 2 have been deposited at the CCDC under 2352958 and 2352959.†

## Conflicts of interest

The authors declare no conflict of interest.

## Supplementary Material

QI-011-D4QI01102C-s001

QI-011-D4QI01102C-s002
