# Supplementary material for: Supramolecular delivery of dinuclear ruthenium and osmium MCU inhibitors
Source: Inorg Chem Front. 2024 Jul 11;11(16):5064–79. doi: 10.1039/d4qi01102c (PMC11301636; doi:10.1039/d4qi01102c)
Supplement: QI-011-D4QI01102C-s001 [file QI-011-D4QI01102C-s001.pdf]

Electronic Supplementary Material (ESI) for Inorganic Chemistry Frontiers.
This journal is © the Partner Organisations 2024

Electronic Supplementary Information for

Supramolecular Delivery of Dinuclear Ruthenium and Osmium MCU Inhibitors

Nicholas P. Bigham,[1] Robyn J. Novorolsky,[2,3] Keana R. Davis,[5] Haipei Zou,[1] Samantha N. MacMillan,[1] Michael J. Stevenson,[5] George S. Robertson,[2,3,4] and Justin J. Wilson[1]*

[1] Department of Chemistry and Chemical Biology, Cornell University, Ithaca, NY, 14853, USA

[2] Department of Pharmacology, Faculty of Medicine, Dalhousie University, Life Sciences Research Institute, Halifax, NS B3H 0A8, Canada

[3] Brain Repair Centre, Faculty of Medicine, Dalhousie University, Life Sciences Research Institute, Halifax, NS, B3H 0A8, Canada

[4] Department of Psychiatry, Faculty of Medicine, Dalhousie University, Life Sciences Research Institute, Halifax, NS, B3H 0A8, Canada

[5] Department of Chemistry, University of San Francisco, San Francisco, CA, 94117, USA

*E-mail: jjw275@cornell.edu

Table of Contents

General Considerations	S2
Synthesis and Characterization	S3
Encapsulation Studies and Binding Constant Determination	S13
Aquation Kinetics	S20
Biological Experiments	S22
References	S28

1. General Considerations

Reagents and Materials

All reagents were obtained commercially and used without further purification. Water (18 MΩ·cm) was purified using an EGLA PURELAB flex 2 (High Wycombe, UK). Ru265, Ru265′, Os245, and Os245′ were synthesized and characterized via NMR spectroscopy, IR spectroscopy, and elemental analysis according to literature procedures.[1–3]

Physical Measurements

1D-NMR (^{1}H, $^{13}C\{^{1}H\}$, and $^{19}F\{^{1}H\}$) and 2D-NMR (DOSY) spectra were acquired at 25 °C on a 500 MHz Bruker AV 3HD spectrometer equipped with a broadband Prodigy cryoprobe (Bruker, Billerica, MA). UV-vis spectra were recorded using 1 cm quartz cuvettes and a Shimadzu UV-1900 spectrometer (Shimadzu, Kyoto, Japan) fitted with a temperature-controlled circulating water bath. IR spectra were recorded using a Bruker Tensor II Infrared Spectrometer equipped with a diamond ATR crystal (Bruker, Billerica, MA). Elemental analyses (C,H,N) were carried out by Atlantic Microlab Inc. (Norcross, GA). ITC experiments were carried out on a TA Instruments Nano ITC (New Castle, DE) at 25.0 ± 0.1 °C with a stir rate of 150 rpm. Each ITC experiment consisted of 20 injections of 2.5 μL titrant and were analyzed using NanoAnalyze software. Fluorescence and absorbance of samples in 96-well plates were measured using a BioTek Synergy HT plate reader (Winooski, VT). GFAAS was performed using a PinAAcle 900Z spectrometer (Perkin Elmer, Waltham, MA). Standardized solutions (0–200 μg L^{-1}) of ruthenium, which were prepared from a standardized ICP-MS solution of Ru (1001 ± 4 μg L^{-1} in 10 % HCl, Agilent Technologies, Santa Clara, CA) were used to generate a calibration curve. ICP-MS was performed using an Agilent ICP-MS 7800 fitted with a Micromist nebulizer with a sample uptake rate of 0.4 mL min^{-1}. Ni was used as the cone material. The Os content was determined by tracking ^{189}Os and a series of standardized solutions containing 0–200 μg L^{-1} Os, which were prepared from a standardized ICP-MS solution of Os (1000 ± 5 μg mL^{-1} in 20% HCl, Agilent Technologies, Santa Clara, CA). All calibration standards and samples were prepared in a stabilizing solution containing 500 μM each of ethylenediaminetetraacetic acid (EDTA), thiourea, and ascorbic acid to prevent the loss of Os as volatile OsO_4 under the oxidizing conditions required for ICP-MS.[4] Concentrations of all ruthenium and osmium stock solutions applied in analytical and biological experiments were verified by GFAAS or ICP-MS, respectively. Statistical analyses were performed using GraphPad Prism version 9.5.1 for Mac OS (GraphPad Software, San Diego, CA) by applying a non-paired student's t-test. Curve fitting was also performed using GraphPad Prism. All studies involving animals were conducted in agreement with the guidelines set by the Canadian Council on Animal Care (CCAC) and approved by the University Committee on Laboratory Animals (UCLA) at Dalhousie University.

2. Synthesis and Characterization

Synthesis of $NaO_2CC_{10}H_{15}$ (NaOAd)

To a 1 M NaOH solution in ethanol (5.55 mL, 5.55 mmol) was added 1-adamantane carboxylic acid (1.00 g, 5.55 mmol), affording a white suspension that upon stirring 1 h at room temperature became translucent and colorless. The solution was then concentrated to dryness to a white powder and dried in vacuo. Yield: 1.05 g (93.8%, 5.19 mmol). ^{1}H NMR (500 MHz, DMSO-*d*$_6$) δ (ppm) = 1.88 (m, 6 H), 1.71 (d, 12 H), 1.61 (dd, 12 H). ^{13}C{^{1}H} NMR (126 MHz, DMSO-*d*$_6$) δ (ppm) = 181.04, 40.31, 40.20, 36.92, 28.24. IR (ATR) ν (cm^{-1}) = 3358 (m, br), 2905 (s), 2842 (m), 1540 (m), 1519 (s), 1417 (m), 1398 (s), 1313 (m), 908 (w), 804 (w), 761 (w), 681 (m), 592 (m, br). Elemental analysis (C,H,N): calc'd (%, for $NaC_{11}H_{15}O_2 \cdot H_2O$) C 55.99; H 7.78; N 0. Found (%) C 60.21, H 7.91, N 0.12.

Synthesis of $1 \cdot 2H_2O \cdot NaCF_3SO_3$

The triflate ($CF_3SO_3^-$) salt of Ru265′ (100 mg, 0.088 mmol) was dissolved in 10 mL methanol with stirring. A solution of NaOAd (35.6 mg, 0.176 mmol) in 5 mL methanol was added to the orange Ru265′ solution dropwise with stirring. The clear, orange solution was then heated at 60 °C for 12 h. The solution was then concentrated to dryness, and the remaining orange residue was redissolved in ~3 mL THF. The room temperature vapor diffusion of hexanes over a period of ~2 d into this THF solution afforded orange crystals. These crystals were collected, washed sequentially with 10 mL ethanol, 10 mL acetone, and 10 mL diethyl ether, and then dried in vacuo for 12 h. Yield: 96.3 mg (80.0%, 0.070 mmol) ^{1}H NMR (500 MHz, DMSO-*d*$_6$) δ (ppm) = 3.90 (s, 24 H), 1.91 (m, 6 H), 1.71 (d, 12 H), 1.63 (dd, 12 H). ^{13}C{^{1}H} NMR (126 MHz, DMSO-*d*$_6$) δ (ppm) = 185.56, 124.41–116.72 ($CF_3SO_3^-$), 41.10, 39.16, 36.34, 27.72. ^{19}F{^{1}H} NMR (470 MHz, DMSO-*d*$_6$) δ (ppm) = –77.75. IR (ATR) ν (cm^{-1}) = 3323 (m, br), 2905 (s), 2849 (m), 1555 (m), 1378 (m), 1256 (s), 1170 (s), 1049 (s), 1025 (s), 763 (m), 678 (m), 638 (s), 562 (m), 520 (m). Elemental analysis (C,H,N): calc'd (%, for $C_{25}H_{54}N_9O_{13}S_3F_9Ru_2 \cdot 2H_2O \cdot NaCF_3SO_3$) C 22.86; H 4.28; N 9.23. Found (%) C 22.42, H 4.46, N 8.98.

Synthesis of $2 \cdot 2H_2O \cdot NaCF_3SO_3$

The triflate ($CF_3SO_3^-$) salt of Os245′ (80 mg, 0.060 mmol) was dissolved in 10 mL methanol with stirring. A solution of NaOAd (24.4 mg, 0.120 mmol) in 5 mL methanol was added to the yellow Os245′ solution dropwise with stirring. The clear, yellow solution was heated at 50 °C for 48 h. The solution was then concentrated to dryness and redissolved in ~3 mL THF. This solution was placed in a vial and was slowly evaporated at 4 °C over a period of ~5 d until yellowish brown crystals formed. These crystals were collected, washed with 10 mL ethanol, 10 mL acetone, and 10 mL diethyl ether, followed by drying in vacuo for 12 h. Yield: 78.3 mg (83.2%, 0.051 mmol). ^{1}H NMR (500 MHz, DMSO-*d*$_6$) δ (ppm) = 4.74 (s, 24 H), 1.91 (m, 6 H), 1.71 (d, 12 H), 1.63 (dd, 12 H). ^{13}C{^{1}H} NMR (126 MHz, DMSO-*d*$_6$) δ (ppm) = 185.39, 124.41–116.72 ($CF_3SO_3^-$), 40.78, 38.38, 36.33, 27.69. ^{19}F{^{1}H} NMR (470 MHz, DMSO-*d*$_6$) δ (ppm) = –77.75. IR (ATR) ν (cm^{-1}) = 3309 (m, br), 2912 (s), 2857 (m), 1681 (m), 1389 (m), 1264 (s), 1173 (s), 1116 (s), 1027 (s), 768 (m), 683 (m), 634 (s), 577 (m), 522 (m). Elemental analysis (C,H,N): calc'd (%, for

$C_{25}H_{54}N_9O_{13}S_3F_9Os_2{\cdot}2H_2O{\cdot}NaCF_3SO_3$) C 20.22; H 3.79; N 8.16. Found (%) C 20.27, H 3.90, N 8.31.

Synthesis of Cucurbit-[7]-uril (CB[7])

This procedure was adapted from a literature procedure.[5] Glycoluril (3.00 g, 21 mmol) was added to 3 mL concentrated HCl and stirred at room temperature. Slowly, paraformaldehyde (1.35 g, 45 mmol) was added, and the cloudy, white mixture was heated at 100 °C for 12 h. The solution, now orange and clear, was added to 25 mL H_2O and stirred at room temperature for 1 h. A white precipitate, presumably a mixture of CB[6] and CB[8], formed and was removed by filtration. The remaining clear, orange filtrate was collected, and 25 mL methanol was added. A white precipitate formed immediately, which was filtered and washed with an additional 15 mL methanol. This white solid, a mixture of CB[5] and CB[7], was next added to 32 mL 20% (v/v) glycerol in H_2O and heated at 100 °C for 4 h. The remaining white precipitate was filtered and dried as crude CB[5]. The filtrate, containing the desired CB[7] product, was heated at 110 °C for 2 h or until the mixture became viscous. The thick mixture was added to 50 mL stirring methanol and continued stirring for 12 h at room temperature. The final white precipitate in this mixture was filtered, washed with 25 mL methanol and 25 mL diethyl ether, and dried. Yield: 257 mg (7.6%, 0.221 mmol). ^1H NMR (500 MHz, D_2O + 0.1% DCl) δ (ppm) = 5.78 (d, 14 H), 5.53 (s, 14 H), 4.24 (d, 14 H). $^{13}C\{^1H\}$ NMR (126 MHz, D_2O + 0.1% DCl) δ (ppm) = 156.68, 71.25, 52.62. IR (ATR) ν (cm^{-1}) = 3449 (m, br), 1722 (s), 1471 (s), 1372 (m), 1317 (m), 1230 (s), 1187 (s), 966 (s), 796 (s), 670 (m). Elemental analysis (C,H,N): calc'd (%, for CB[7]$\cdot 16H_2O$) C 34.76; H 5.14; N 27.03. Found (%) C 34.43, H 5.19, N 26.94.

X-Ray Crystallography

Single crystals of **1** were obtained via the vapor diffusion of *n*-hexane into a solution of the compound in THF at room temperature. Single crystals of **2** were grown through the evaporation of a THF solution of the compound at 4 °C. Low-temperature X-ray diffraction data for **1** and **2** were collected on a Rigaku XtaLAB Synergy diffractometer coupled to a Rigaku Hypix detector with Cu Kα radiation (λ = 1.54184 Å) from PhotonJet micro-focus X-ray sources at 100 K. The diffraction images were processed and scaled using the CrysAlisPro software (Rigaku Oxford Diffraction, The Woodlands, TX). The structures were solved through intrinsic phasing using SHELXT[6] and refined against F^2 on all data by full matrix least squares with SHELXL[7] following established refinement strategies.[8] All non-hydrogen atoms were refined anisotropically. Hydrogens atoms on the complex cations of **1** and **2** were added with a riding model (HFIX 137, HFIX 23, or HFIX 13). The hydrogen atoms on the solvent water molecules could not be located on the difference map and were therefore not included in the model. This omission led to B-level CheckCIF alerts. However, based on charge balance considerations and putative hydrogen-bonding interactions within the crystal structure, the hydrogen atoms are present to afford neutral water molecules. Both structures were isomorphic, and their asymmetric unit comprised half of the complex cations of **1** and **2**, which resided on crystallographic inversion centers, a THF molecule, two triflate anions, and residual electron density peaks. Charge balance considerations required the presence of an additional cation within the unit cell. Accordingly, residual electron density could be satisfactorily modeled as a half-occupancy sodium atom with site position disorder with a

lattice water molecule with occupancy factors fixed at 0.50 for each component. Within **1**, both of the outer-sphere triflate ions exhibited conformational disorder, which was modeled using appropriate similarity restraints. In addition, the adamantyl moiety of this complex was also disordered over two rotational orientations, which were modeled with similarity restraints. For **2**, one of the triflate ions had elongated ellipsoids, indicating unresolved disorder, which triggered B-level alerts in the CheckCIF. Based on our refinement model, the final formula unit within each unit cell was $[(C_{10}H_{15}CO_2)Os(NH_3)_4(\mu\text{-}N)Os(NH_3)_4(O_2CC_{10}H_{15})](CF_3SO_3)_3\cdot NaCF_3SO_3\cdot THF\cdot 3H_2O$. CCDC 2352958–2352959 contain the supplementary crystallographic data for this paper. These data can be obtained free of charge via www.ccdc.cam.ac.uk/data_request/cif, or by emailing data_request@ccdc.cam.ac.uk, or by contacting The Cambridge Crystallographic Data Centre, 12 Union Road, Cambridge CB2 1EZ, UK; fax: +44 1223 336033.

Table S1. X-ray crystal data and structure refinement details for **1** and **2**.

**Compound**	**1**	**2**
Empirical formula	$C_{34}H_{70}F_{12}N_9NaO_{22}Ru_2S_4$	$C_{34}H_{70}F_{12}N_9NaO_{21}Os_2S_4$
Formula weight	1522.36	1700.62
a (Å)	8.88170(10)	8.91090(10)
b (Å)	31.0392(2)	31.1434(2)
c (Å)	10.81930(10)	10.81940(10)
α (°)	90	90
β (°)	98.3730(10)	98.3470(10)
γ (°)	90	90
V (Å^3)	2950.88(5)	2970.75(5)
Z	2	2
Crystal system	Monoclinic	Monoclinic
Space group	$P2_1/c$	$P2_1/c$
ρ_{calcd} (Mg m^{-3})	1.713	1.901
μ (Cu Kα), mm^{-1} [a]	6.577	10.370
λ (Å)	1.54184	1.54184
T (K)	100.3(9)	103.4(6)
2θ range (°)	2.847–77.944	2.835–77.711
Independent reflections	6264	6317
R_{int}	0.0488	0.0571
Number of parameters	541	389
Largest diff. peak and hole (eÅ^{-3})	1.483/–1.079	2.743/–2.242
GoF[b]	1.027	1.051
R_1/wR^2 (all data)[c]	0.0622/0.1521	0.0604/0.1441
R_1/wR^2 ($I > 2\sigma$)[c]	0.0595/0.1505	0.0571/0.1415

[a]Cu Kα λ = 1.54184 Å

[b]GoF = $\{\Sigma(w(F_o^2 - F_c^2)^2/(n - p)\}^{1/2}$, where *n* = number of data and *p* is the number of refined parameters.

[c]$R_1 = \Sigma||F_o| - |F_c||/\Sigma|F_o|$; $wR^2 = \{\Sigma[w(F_o^2 - F_c^2)^2]/\Sigma[(F_o^2)^2]\}^{1/2}$

Table S2. Selected interatomic distances and angles for **1** and **2**.[b]

Selected interatomic distances (Å) for **1•2H_2O•THF**				Selected interatomic distances (Å) for **2•2H_2O•THF**			
Ru–O(1)	2.093(3)	Ru–N(3)	2.102(4)	Os–O(1)	2.105(5)	Os–N(3)	2.113(6)
Ru–N(1)	2.108(4)	Ru–N(4)	2.104(4)	Os–N(1)	2.111(6)	Os–N(4)	2.122(7)
Ru–N(2)	2.098(4)	Ru–N(5)	1.7424(3)	Os–N(2)	2.111(6)	Os–N(5)	1.7685(3)
**Selected interatomic angles (°) for 1•2H_2O•THF**				**Selected interatomic angles (°) for 2**			
N(5)–Ru–O(1)	176.44(9)	N(1)–Ru–O(1)	88.99(14)	N(5)–Os–O(1)	176.52(14)	N(1)–Os–O(1)	89.3(2)
N(5)–Ru–N(1)	93.70(11)	N(2)–Ru–N(3)	90.0(2)	N(5)–Os–N(1)	93.46(18)	N(2)–Os–N(3)	90.4(4)
N(5)–Ru–N(2)	95.17(13)	N(2)–Ru–N(4)	169.20(18)	N(5)–Os–N(2)	95.2(2)	N(2)–Os–N(4)	168.8(3)
N(5)–Ru–N(3)	95.68(14)	N(2)–Ru–O(1)	87.19(16)	N(5)–Os–N(3)	95.4(2)	N(2)–Os–O(1)	86.9(3)
N(5)–Ru–N(4)	95.62(12)	N(3)–Ru–N(4)	89.4(2)	N(5)–Os–N(4)	95.9(2)	N(3)–Os–N(4)	89.5(4)
N(1)–Ru–N(2)	89.1(2)	N(3)–Ru–O(1)	81.64(16)	N(1)–Os–N(2)	89.3(3)	N(3)–Os–O(1)	81.8(3)
N(1)–Ru–N(3)	170.62(17)	N(4)–Ru–O(1)	82.05(15)	N(1)–Os–N(3)	171.1(3)	N(4)–Os–O(1)	82.0(3)
N(1)–Ru–N(4)	89.7(2)	Ru–N(5)–Ru#[a]	180.0	N(1)–Os–N(4)	89.1(4)	Os–N(1)–Os#[a]	180.0

[a] # indicates symmetry generated atoms

[b] Atoms are labelled as shown in **Figure 1**.

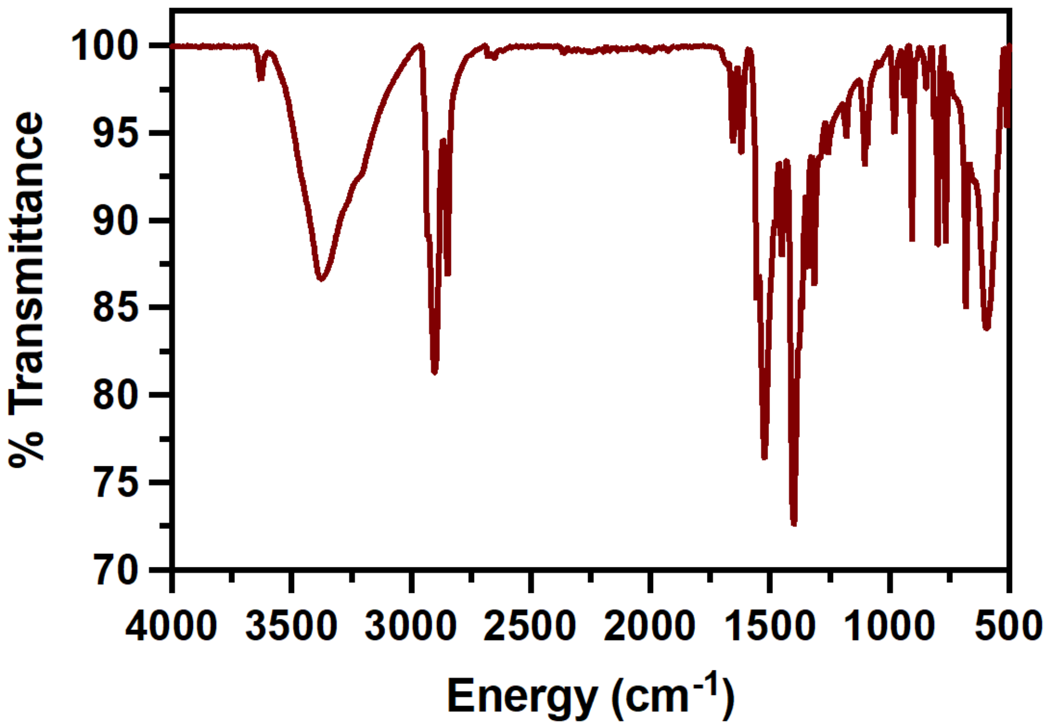

Figure S1. IR (ATR) spectrum of NaOAd.

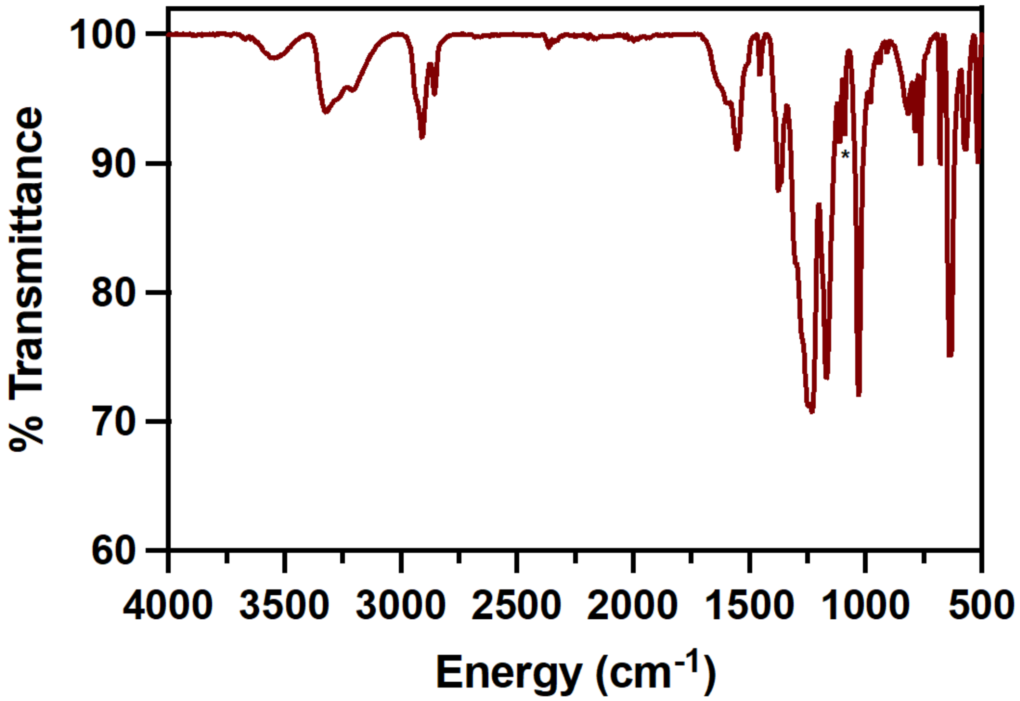

Figure S2. IR (ATR) spectrum of **1**. The asymmetric RuNRu stretch on the IR spectrum is indicated with an asterisk (*).

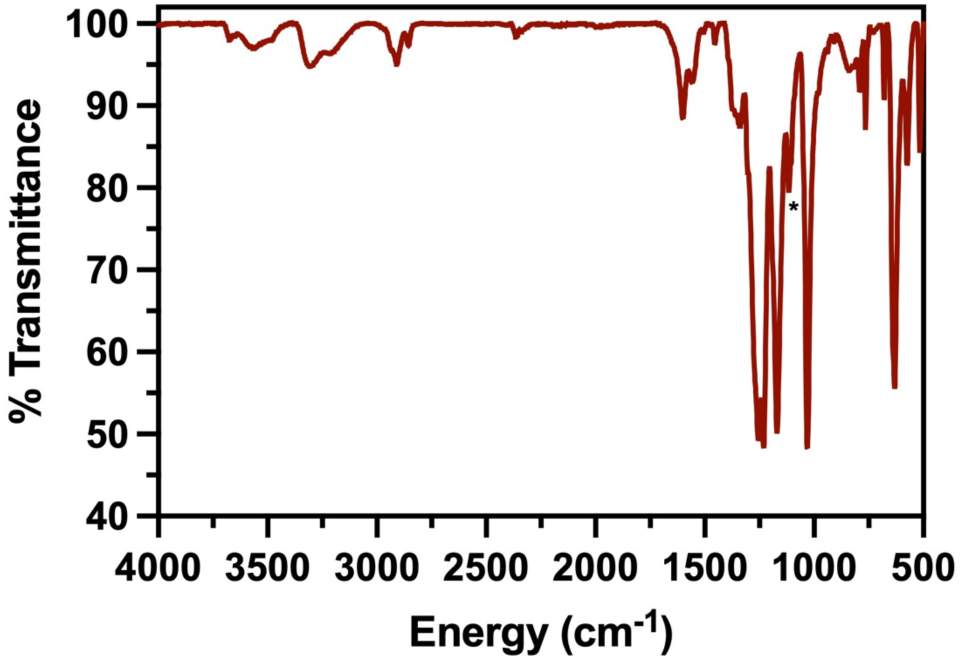

Figure S3. IR (ATR) spectrum of **2**. The asymmetric OsNOs stretch on the IR spectrum is indicated with an asterisk (*).

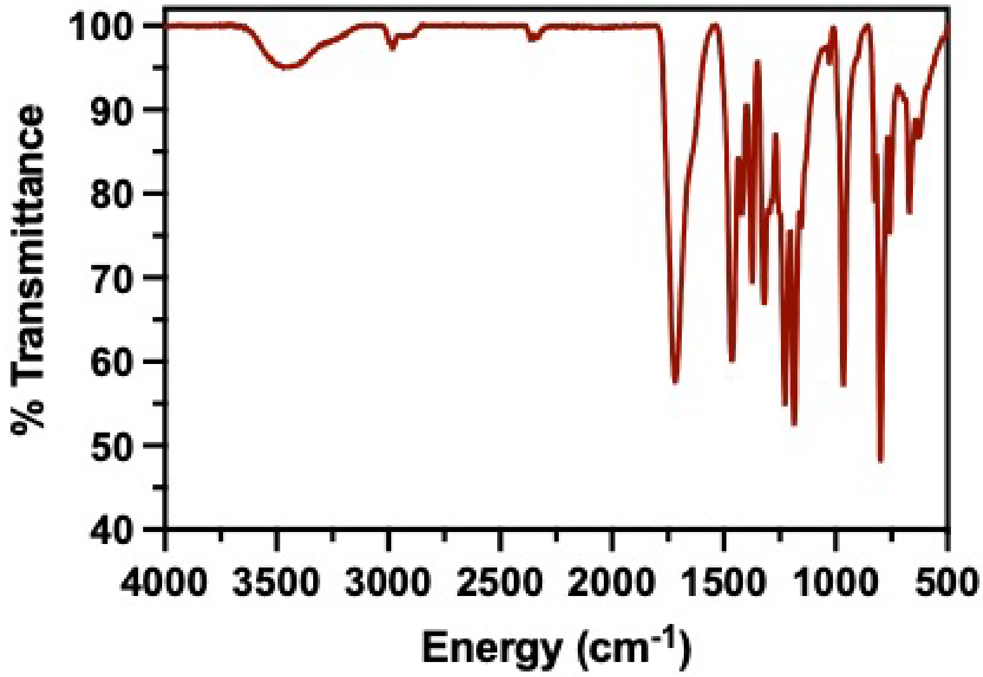

Figure S4. IR(ATR) spectrum of CB[7].

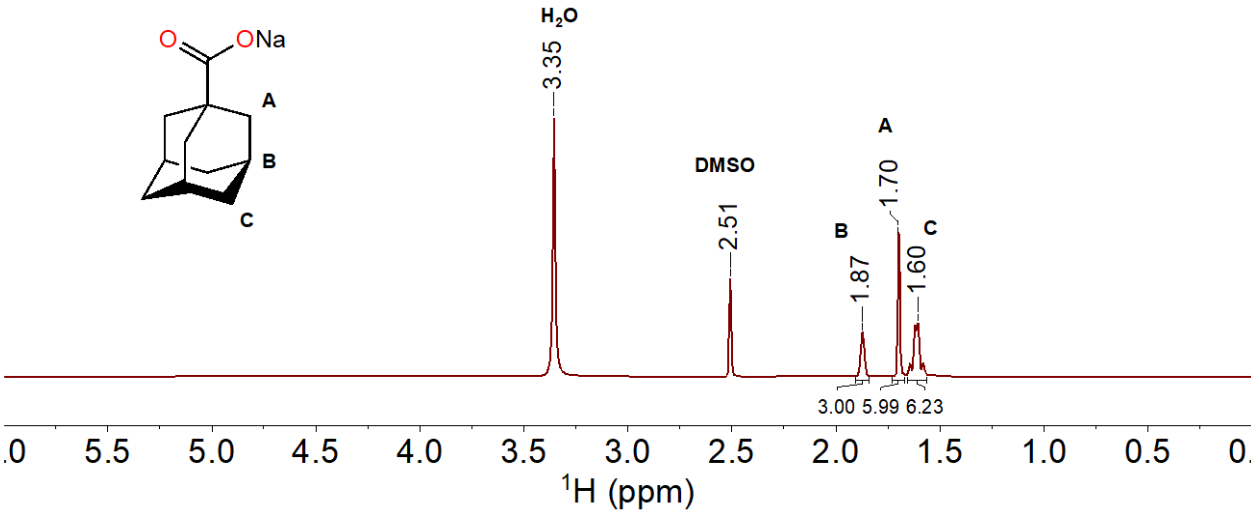

Figure S5. ^{1}H NMR (500 MHz) spectrum of NaOAd in DMSO-d_6 at 25 °C.

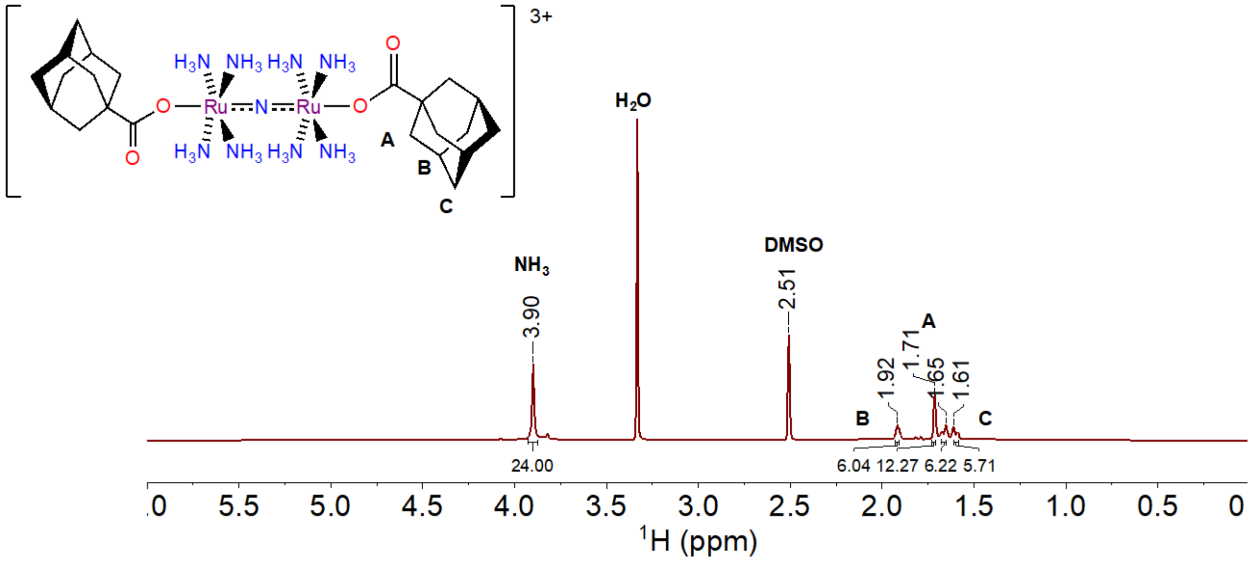

Figure S6. ^{1}H NMR (500 MHz) spectrum of **1** in DMSO-d_6 at 25 °C.

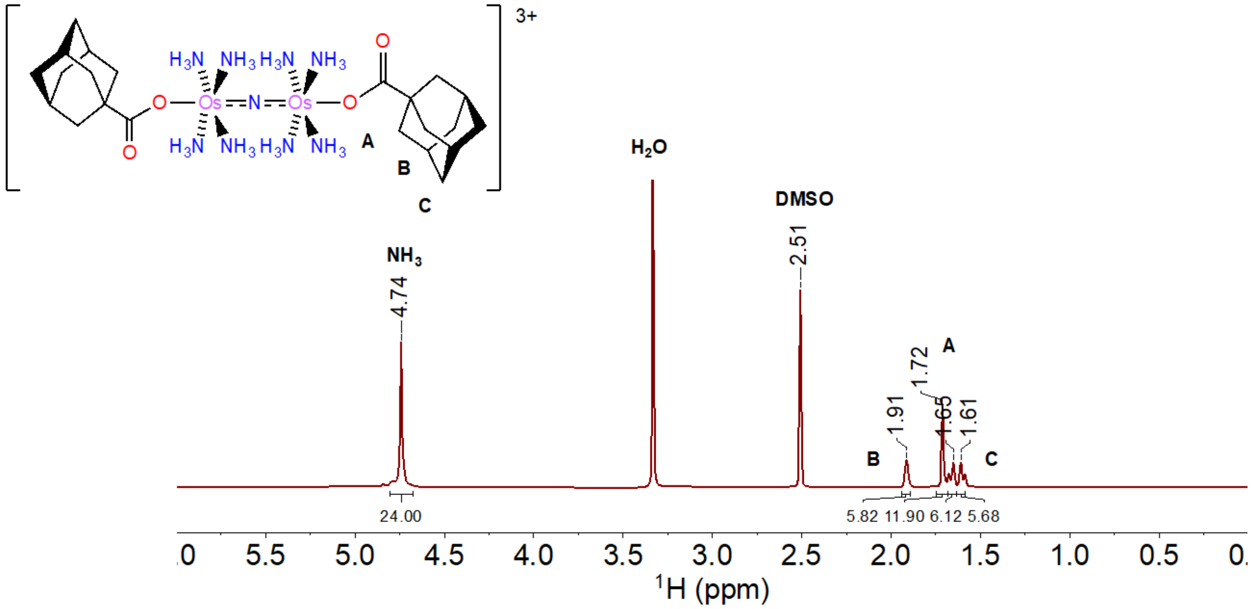

Figure S7. ^{1}H NMR (500 MHz) spectrum of **2** in DMSO-d_6 at 25 °C.

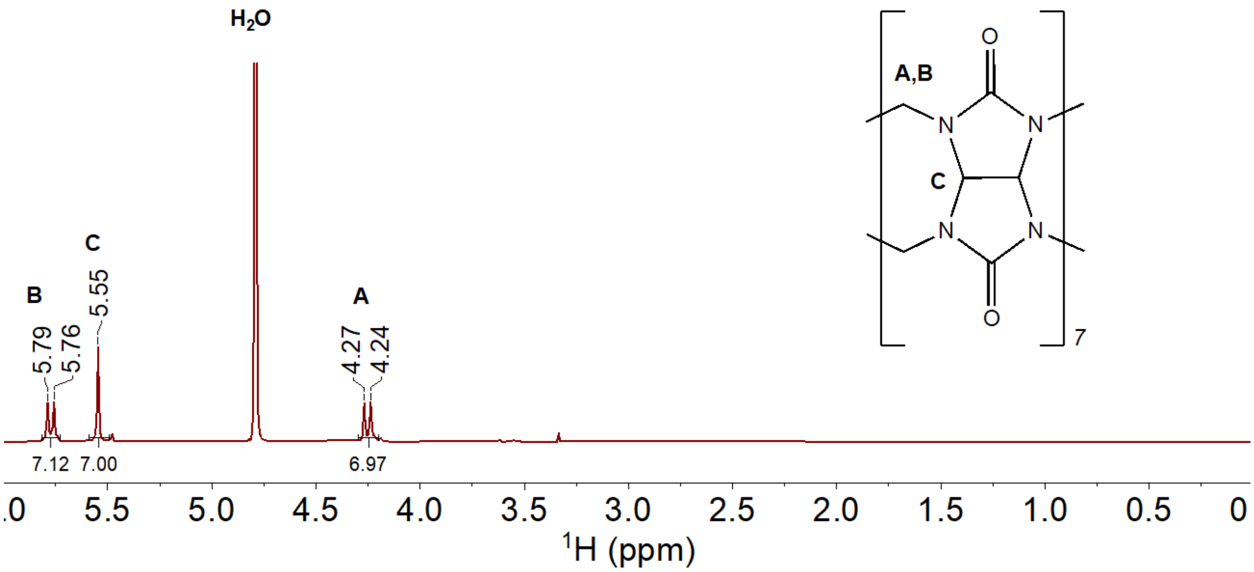

Figure S8. ^{1}H NMR (500 MHz) spectrum of CB[7] in D_2O + 0.1% DCl at 25 °C.

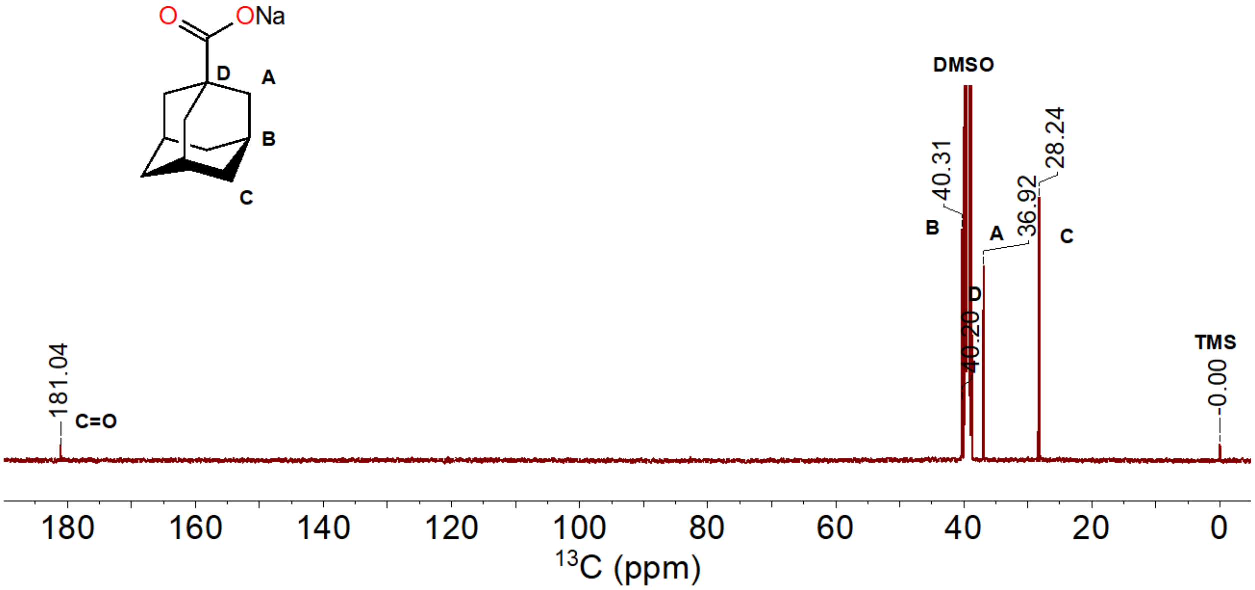

Figure S9. $^{13}C\{^{1}H\}$ NMR (126 MHz) spectrum of NaOAd in DMSO-d_6 at 25 °C.

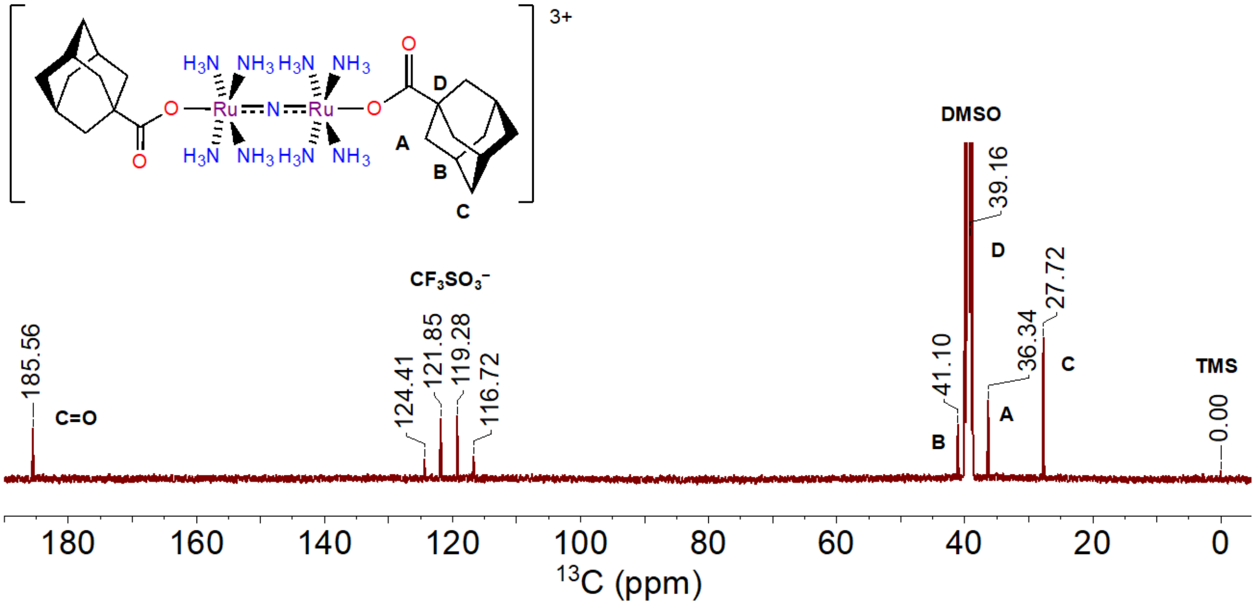

Figure S10. $^{13}C\{^{1}H\}$ NMR (126 MHz) spectrum of **1** in DMSO-d_6 at 25 °C.

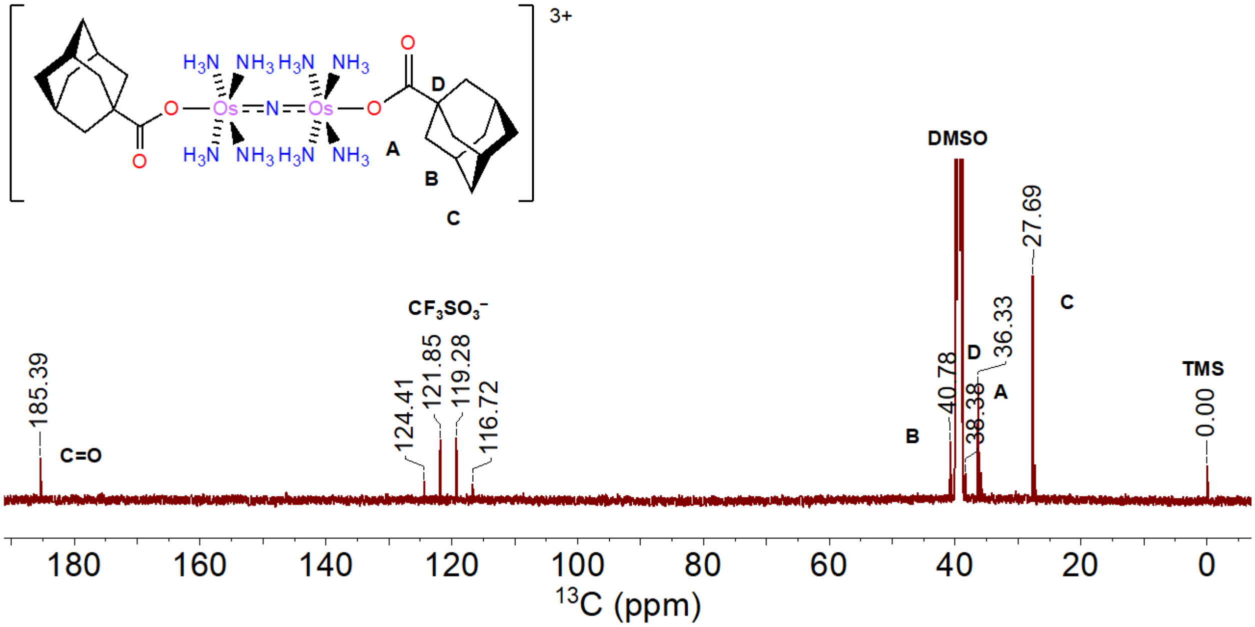

Figure S11. $^{13}C\{^{1}H\}$ NMR (126 MHz) spectrum of **2** in DMSO-d_6 at 25 °C.

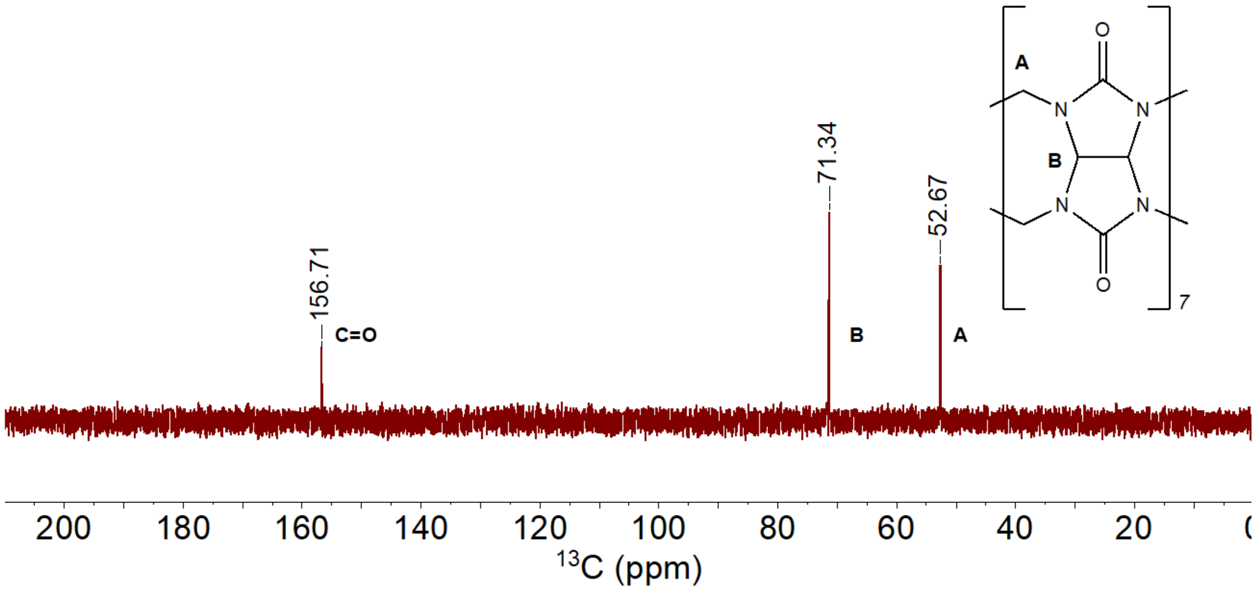

Figure S12. $^{13}C\{^1H\}$ NMR (126 MHz) spectrum of CB[7] in D_2O + 0.1% DCl at 25 °C.

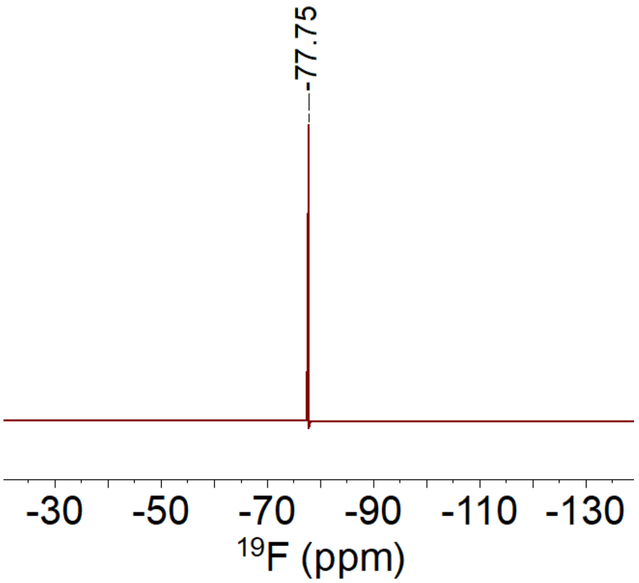

Figure S13. $^{19}F\{^1H\}$ NMR (470 MHz) spectrum of **1** in DMSO-d_6 at 25 °C.

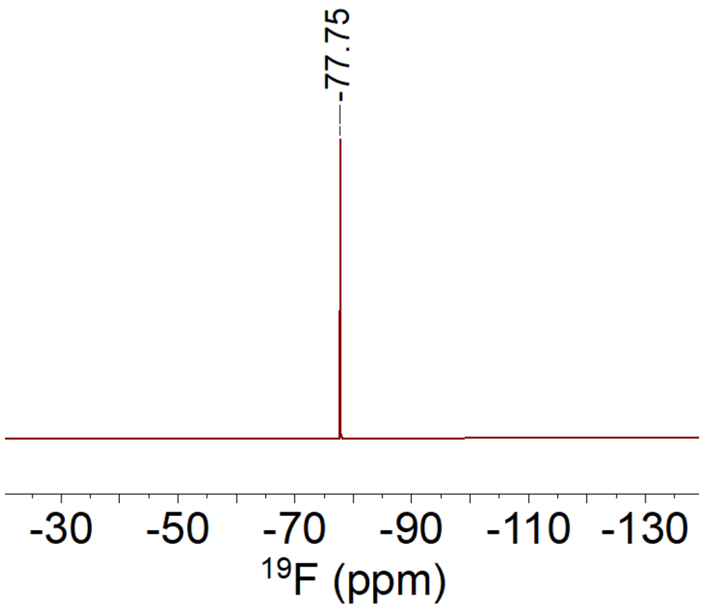

Figure S14. $^{19}F\{^1H\}$ NMR (470 MHz) spectrum of **2** in DMSO-d_6 at 25 °C.

3. Encapsulation Studies and Binding Constant Determination

Encapsulation Studies by ^1H and DOSY NMR

Solutions of **1** (1 mM), **2** (1 mM), or CB[7] (2 mM) were prepared in D_2O (pD = 7). ^1H and DOSY NMR experiments were carried out for **1** or **2** ± CB[7]. The diffusion-ordered experiments were analyzed via the pulse gradient spin-echo experiment. The signal amplitude of each resulting ^1H NMR spectra following the spin echo pulse sequence was fit to **Eqs 1** and **2**. By plotting the natural logarithm of the normalized signal intensities versus the gradient field strength, the slope of the linear fit determines the diffusion constant (D). Results are presented as the average D of three independent experiments ± SD.

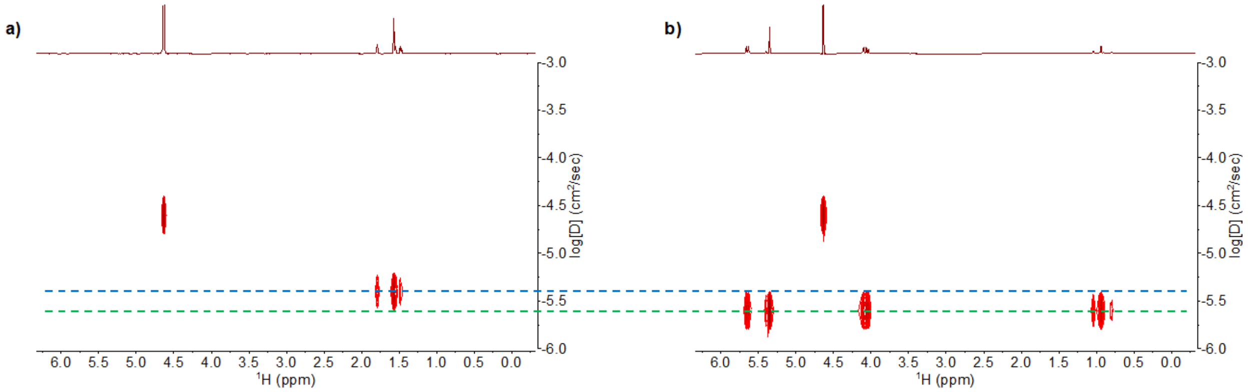

Figure S15. 2D-DOSY NMR (500 MHz) spectra of a) **1** and b) **1** + two equiv. CB[7] at 25 °C. Experiments were performed at 500 µM **1** and 1 mM CB[7].

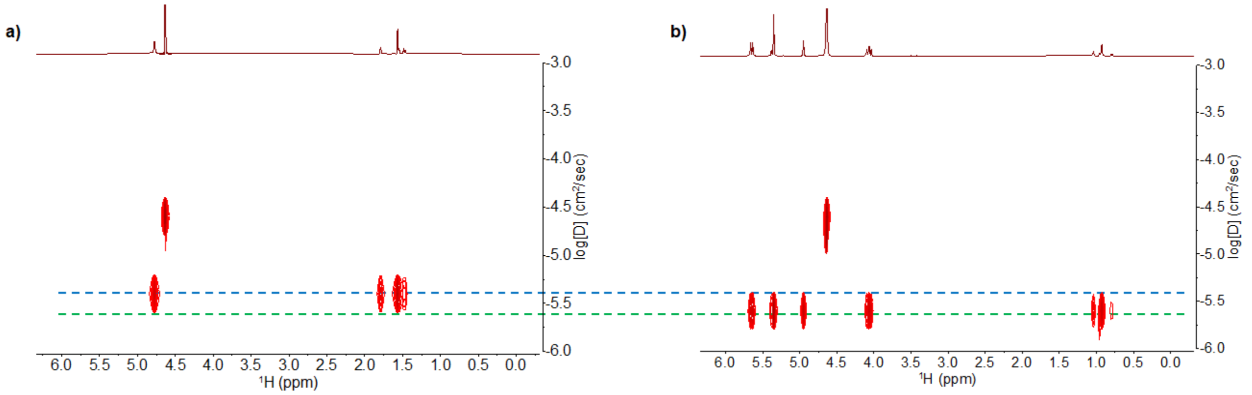

Figure S16. 2D-DOSY NMR (500 MHz) spectra of a) **2** and b) **2** + two equiv. CB[7] at 25 °C. Experiments were performed at 500 μM **2** and 1 mM CB[7].

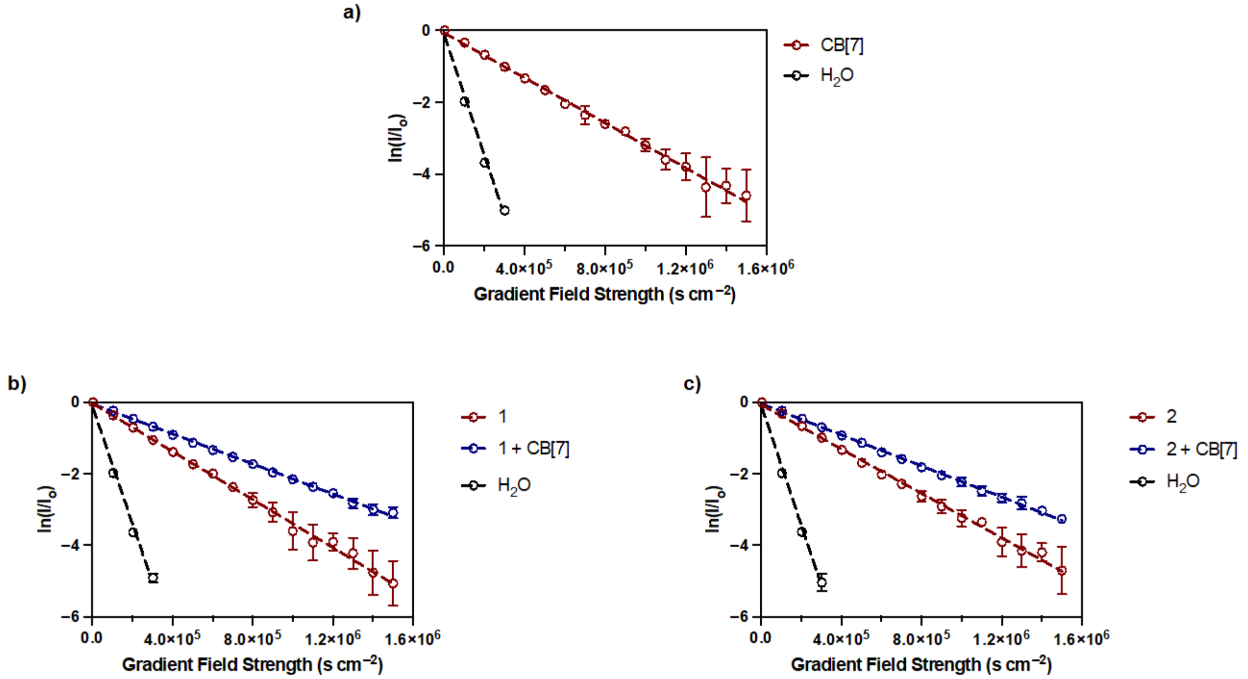

Figure S17. Pulse-gradient spin echo (PGSE) experiment calculations for a) CB[7], b) **1** ± CB[7], and c) **2** ± CB[7]. Integrations of the proton resonances of each species were plotted against the relative gradient field strength (s·cm^{-2}) to measure the diffusion constant (*D*) as the slope of the resulting line. On each plot is shown the plot of H_2O, which consistently measured to be 1.8 × 10^5 cm^2 s^{-1}

Binding Constant Determination by ITC

In these experiments, 50 mM MOPS-buffered solutions (pH 7.4) were used. Prior to buffer preparation, glassware was washed with 10% HNO_3 to remove any trace metals. A 50 mM MOPS solution was prepared and adjusted to pH 7.4 using HCl or NaOH. The buffer was then treated with Chelex 100 resin for at least 1 h while stirring. Prior to storing at room temperature, the buffer was filtered through a 0.22 micron filter. Aliquots for CB[7], NaOAd, **1**, and **2** were prepared by creating a stock solution in this buffer. All aliquots were stored at –20 °C until needed for experimentation. During these experiments, evolution of heat is monitored by measuring the differential power, or heat flow, required to maintain constant temperature within both cells for each injection of the titrant.[9,10] The

resulting thermogram from graphing heat flow (μJ s^{-1}) over time (s) is integrated and normalized for concentration to quantify the stoichiometry (n), binding constant (K_{ITC}), and change in enthalpy (ΔH_{ITC}).[10] Each ITC experiment consisted of 20 injections of 2.5 μL and were analyzed using NanoAnalyze software. Experiments were carried out in triplicate, and the post hoc analysis to determine the K_a for each species was carried out according to literature.[10]

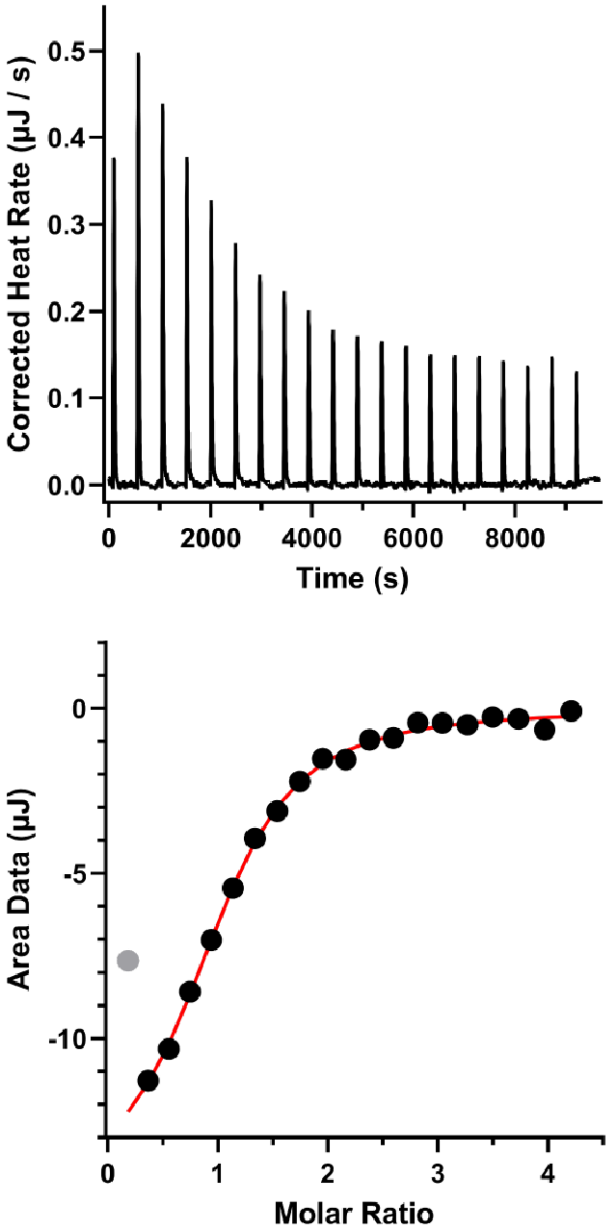

Figure S18. Representative ITC thermogram of 1.0 mM NaOAd titrated into 0.08 mM CB[7] in 50 mM MOPS-buffered solution (pH 7.4). The red line indicates fit from an independent binding model with n = 1.00 ± 0.04, K_{ITC} = (7 ± 1) × 10^4, and ΔH_{ITC} = (–6.0 ± 0.5) kJ mol^{-1}. Grey datum excluded from fit.

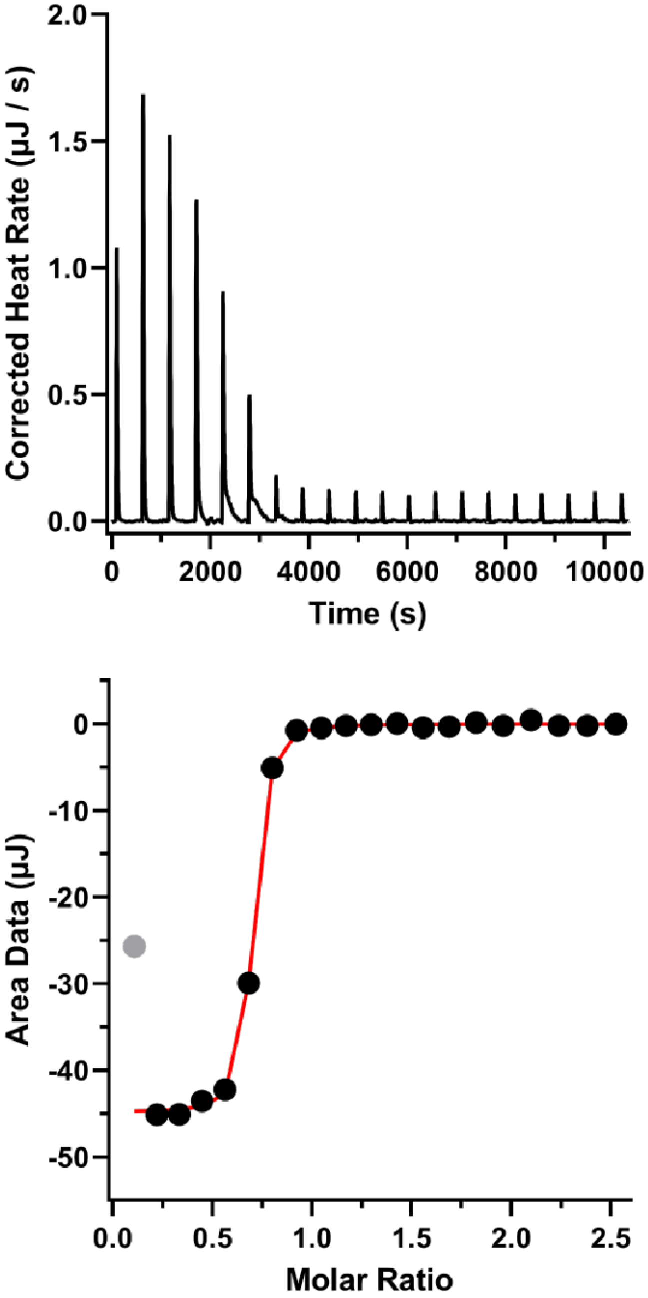

Figure S19. Representative ITC thermogram of 0.6 mM **1** titrated into 0.08 mM CB[7] in 50 mM MOPS-buffered solution (pH 7.4). The red line indicates fit from an independent binding model with n = 0.64 ± 0.08, K_{ITC} = (6 ± 2) × 10^6, and ΔH_{ITC} = (–32.5 ± 0.8) kJ mol^{-1}. Grey datum excluded from fit.

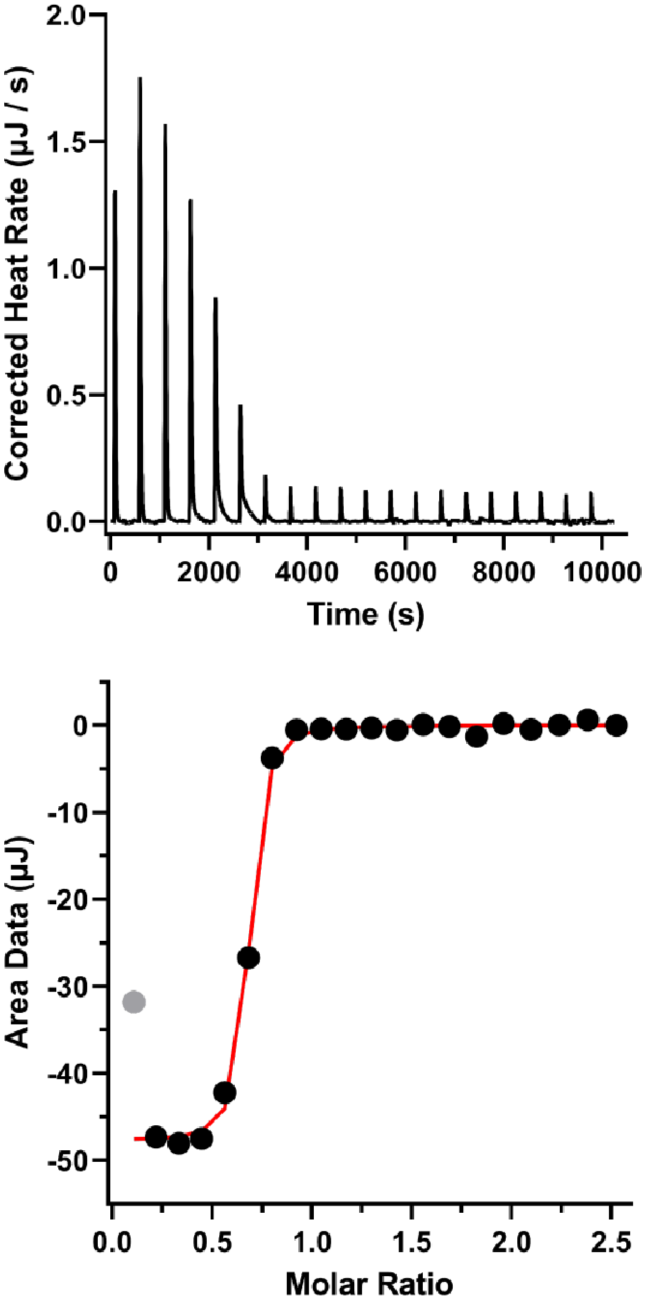

Figure S20. Representative ITC thermogram of 0.6 mM **2** titrated into 0.08 mM CB[7] in 50 mM MOPS-buffered solution (pH 7.4). The red line indicates fit from an independent binding model with $n = 0.65 \pm 0.07$, $K_{ITC} = (9 \pm 1) \times 10^6$, and $\Delta H_{ITC} = (-30.5 \pm 0.3)$ kJ mol^{-1}. Grey datum excluded from fit.

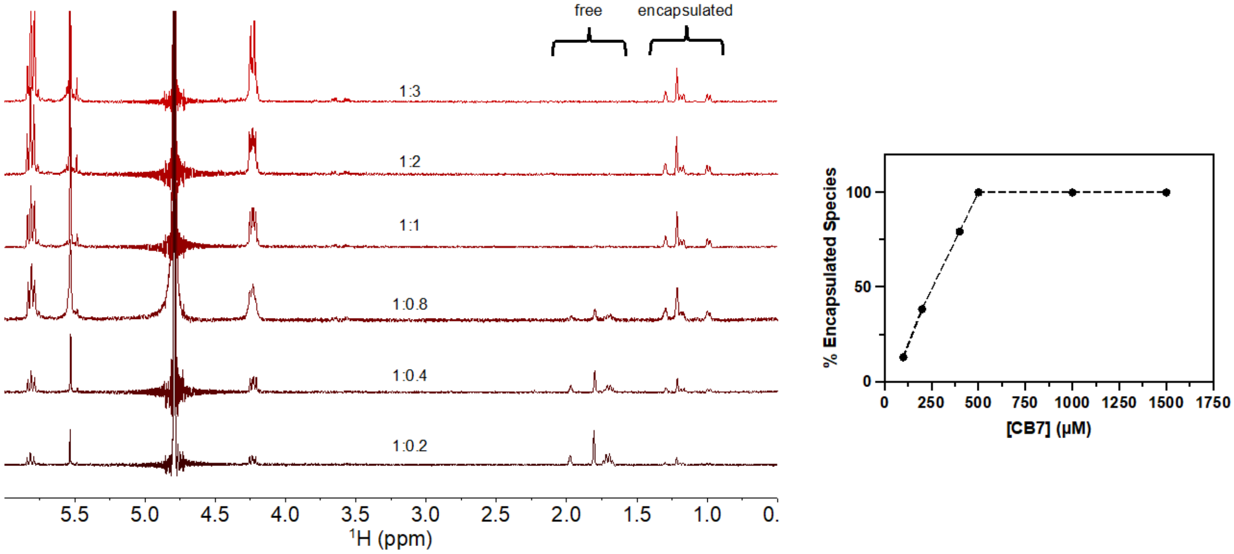

Figure S21. ^{1}H NMR (500 MHz, D_2O, 25 °C) spectra of various concentrations of CB[7] added to 500 μM NaOAd (left). Concentrations of CB[7] range from 125 μM to 1.5 mM. The percent of encapsulated species is plotted against the concentration of CB[7], demonstrating the 1:1 stoichiometry (right).

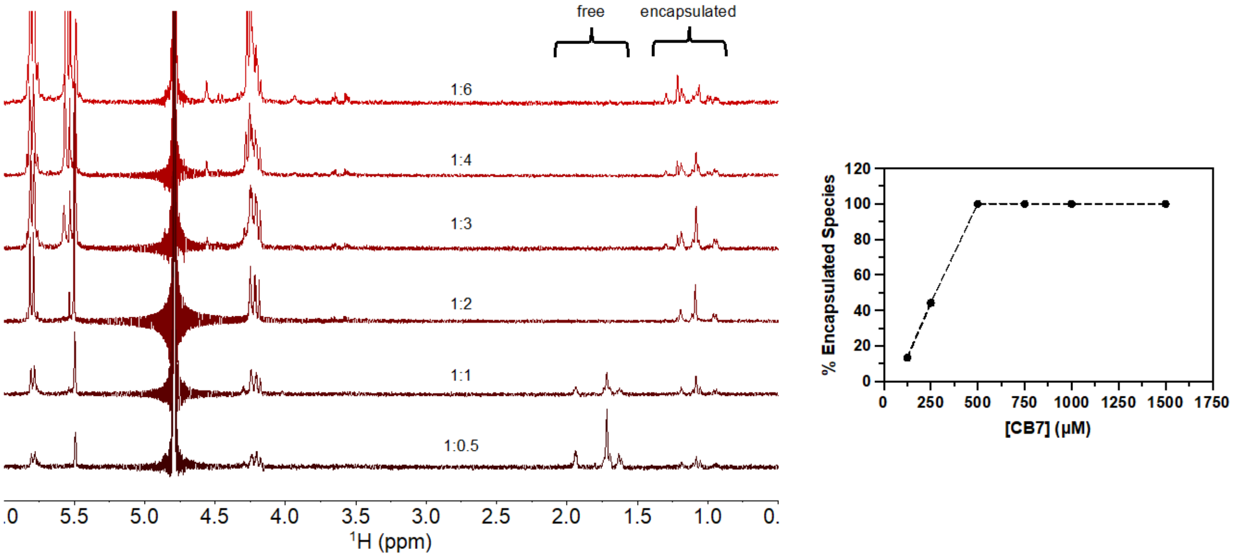

Figure S22. ^{1}H NMR (500 MHz, D_2O, 25 °C) spectra of various concentrations of CB[7] added to 250 μM **1** (left). Concentrations of CB[7] range from 100 μM to 1.5 mM. The percent of encapsulated species is plotted against the concentration of CB[7], demonstrating the 1:2 stoichiometry (right).

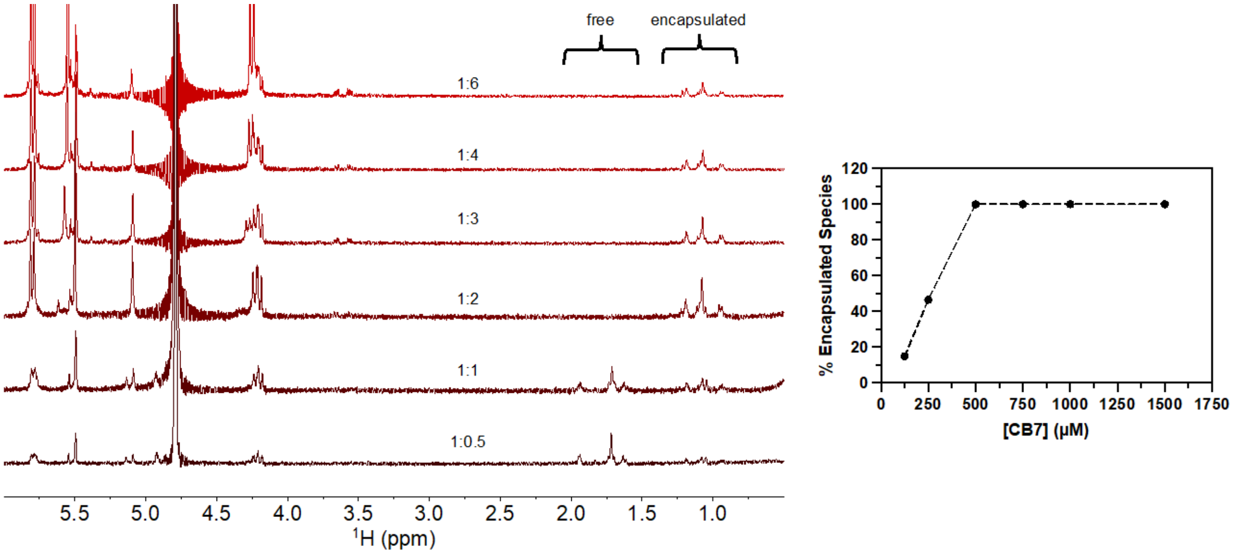

Figure S23. ^{1}H NMR (500 MHz, D_2O, 25 °C) spectra of various concentrations of CB[7] added to 250 μM **2** (left). Concentrations of CB[7] range from 125 μM to 1.5 mM. The percent of encapsulated species is plotted against the concentration of CB[7], demonstrating the 1:2 stoichiometry (right).

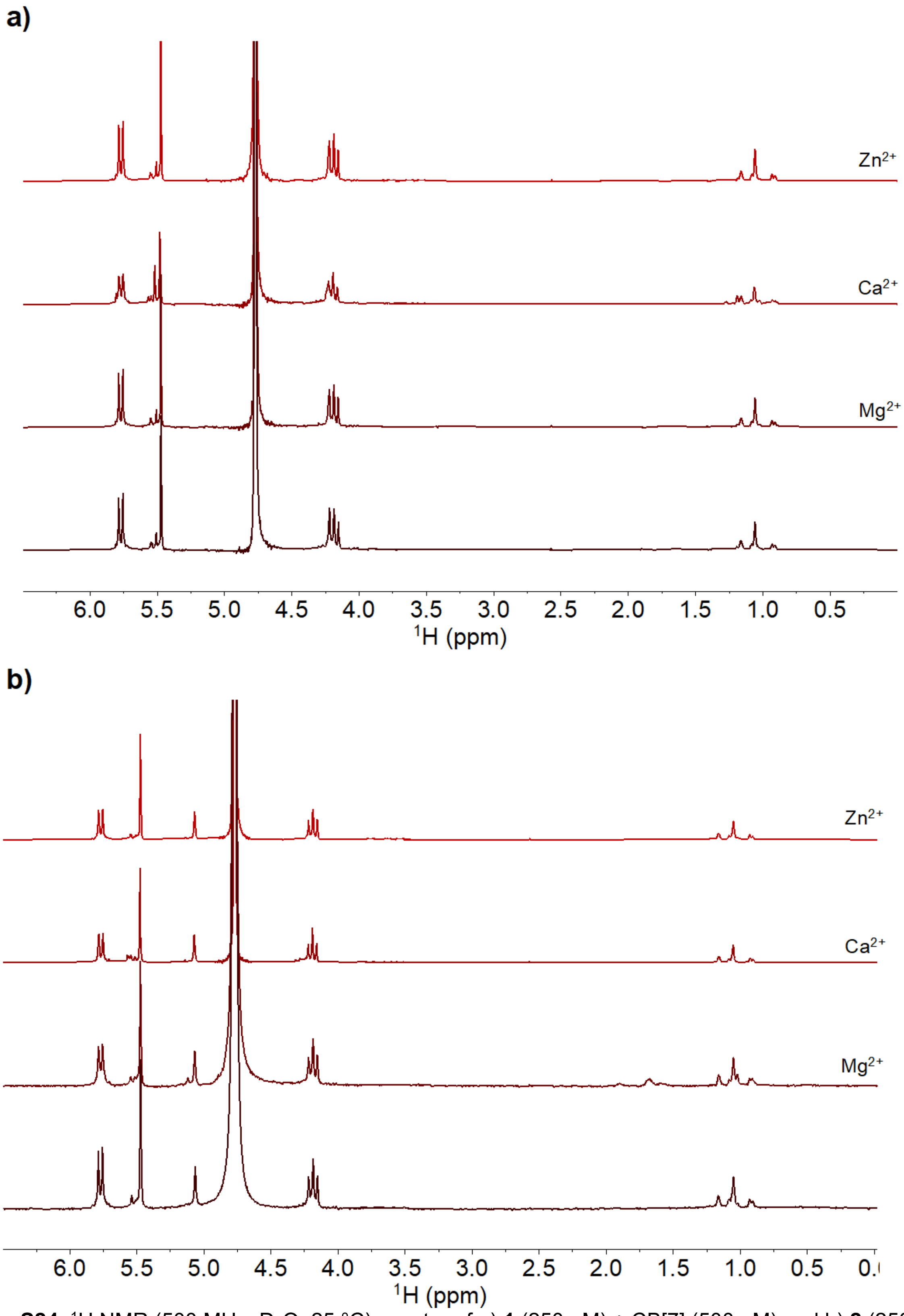

Figure S24. ^{1}H NMR (500 MHz, D_2O, 25 °C) spectra of a) **1** (250 µM) + CB[7] (500 µM) and b) **2** (250 µM) + CB[7] (500 µM) supplemented with $MgCl_2$, $CaCl_2$, or $ZnCl_2$ (2 mM).

4. Aquation Kinetic Studies by UV-vis

Solutions of **1** or **2** (25 μM) with or without CB[7] (0 or 50 μM) in 50 mM MOPS buffered solution (pH 7.4) in a quartz cuvette were placed in a water-jacketed cuvette holder and incubated at 37 °C. The absorbance spectrum of the solution was recorded every 20 min over a period of 16 h for **1** and every 24 h for 120 h for **2**. The pseudo-first order overall rate constant k_{obs} (s^{-1}) was determined by fitting the decrease in absorbance at 261 nm for **1** and 242 nm for **2** as a single exponential decay.

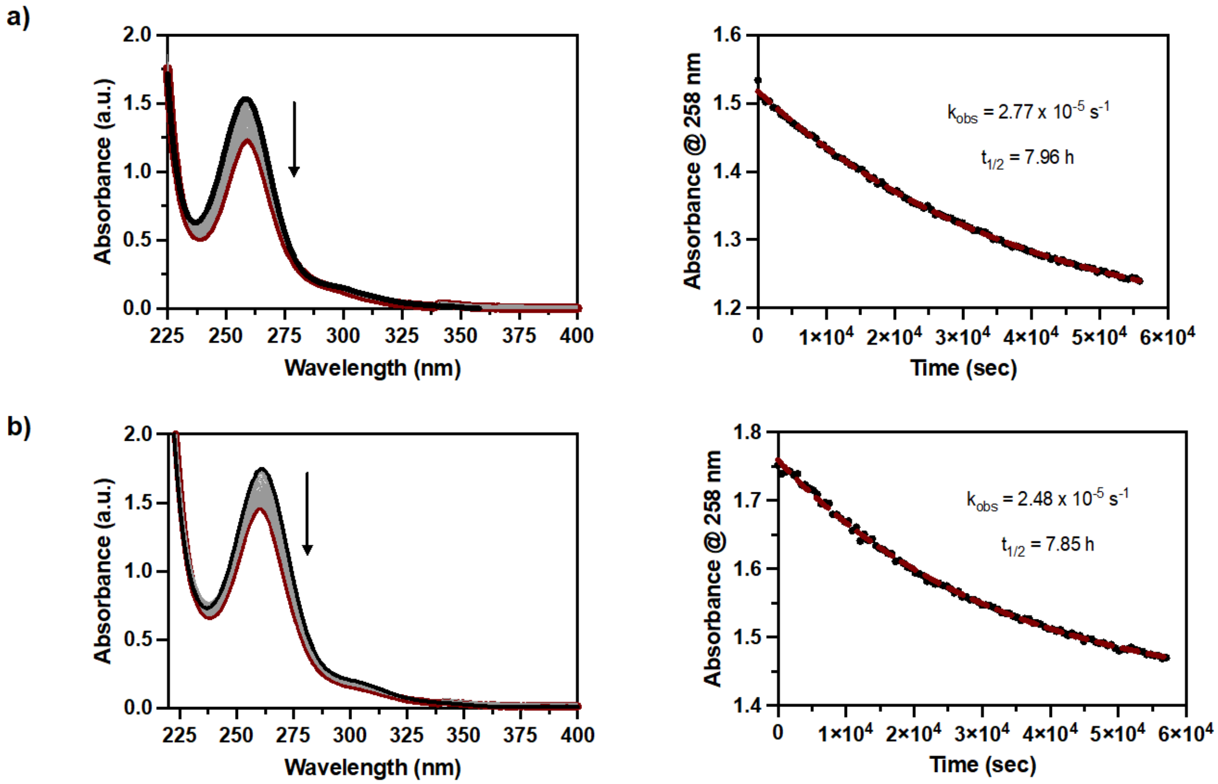

Figure S25. Aquation kinetic experiments of a) **1** and b) **1** + CB[7] in pH 7.4 MOPS buffered solution at 37 °C as measured by UV-vis spectroscopy over 16 h. Shown for each are the change in spectral absorbance at 258 nm over time as indicated by an arrow, and the exponential fit of the absorbance over time to calculate the rate constant of aquation (k_{obs}).

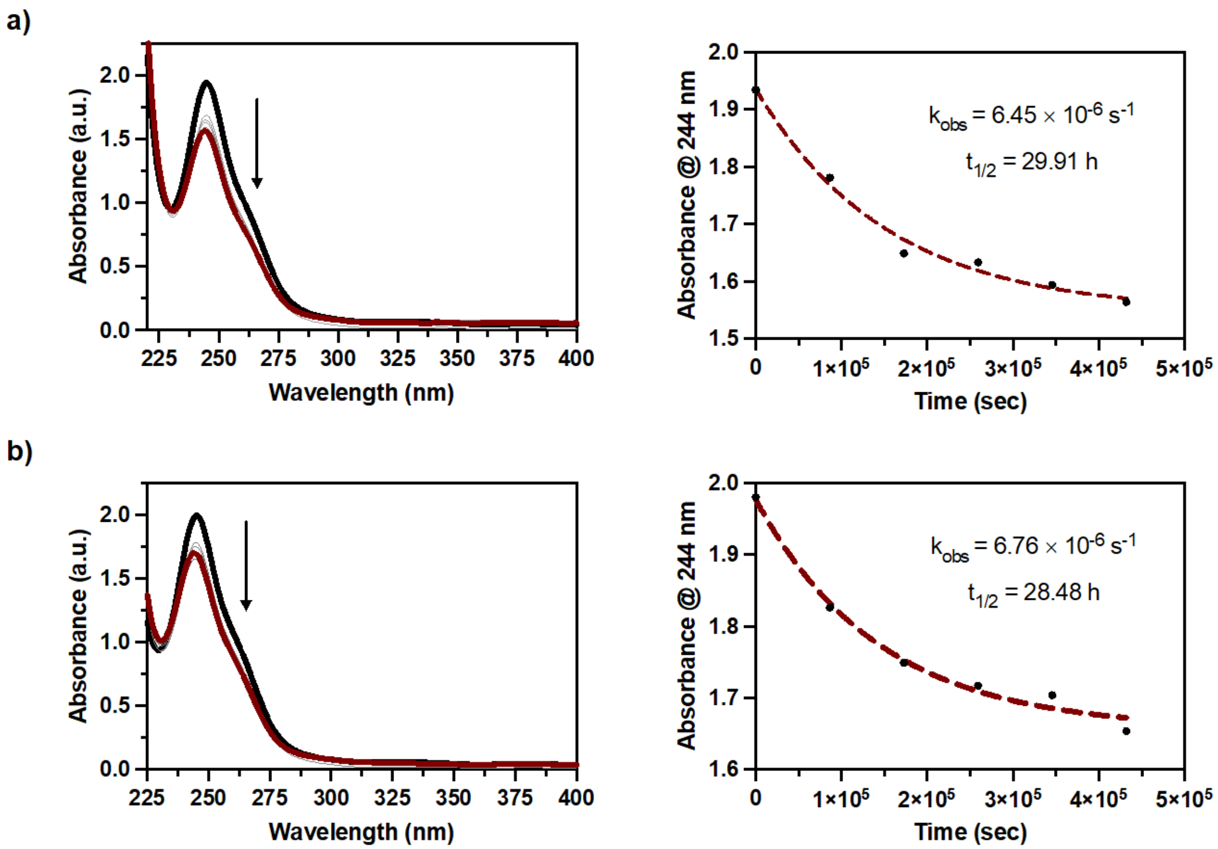

Figure S26. Aquation kinetic experiments of a) **2** and b) **2** + CB[7] in pH 7.4 MOPS buffered solution at 37 °C as measured by UV-vis spectroscopy over 120 h. Shown for each are the change in spectral absorbance at 244 nm over time as indicated by an arrow, and the exponential fit of the absorbance over time to calculate the rate constant of aquation (k_{obs}).

5. Biological Studies

Cytotoxicity

HeLa cells were seeded in 96-well plates with ~4000 cells per well and incubated overnight at 37 °C. On the following day, the culture media was removed, and cells were treated with media containing varying concentrations of the test complex and further incubated for 72 h. The media was removed, and cells were incubated in DMEM containing 1 mg mL^{-1} MTT without FBS for 3 h. Following incubation, the media was removed, and the purple formazan crystals were dissolved in 200 µL of an 8:1 DMSO/glycine buffer (pH 10) mixture. The absorbance of 570 nm of each well was measured using a BioTek Synergy HT plate reader. The average absorbance of control cells was set to 100% viability, and the average absorbances of treated cells were normalized to the control absorbance. Data were plotted as percent viability versus the log[concentration]. The Hill Equation was applied to the data to determine the IC_{50}. Data are reported as the average of three independent biological replicates ± SD.

Mitochondrial Membrane Potential via JC-1 Assay

Approximately 10^5 HeLa cells were seeded in 35 mm glass-bottomed dishes (MatTek Life Sciences, Ashland, MA) and incubated overnight at 37 °C. On the next day, cells were treated with 50 µM of the desired complex and incubated for an additional 24 h at 37 °C. The culture media was then removed and replaced with fresh media supplemented with 10 µM JC-1 dye followed by incubation in the dark for 30 min at 37 °C. The dye-containing media was removed, and the cells were washed with 2 × 1 mL phosphate-buffered saline (PBS, Corning Life Sciences). The cells were imaged in 1 mL PBS. Control dishes were handled identically to treated dishes. For the positive control dishes, 50 µM carbonyl cyanide *m*-chlorophenyl hydrazine (CCCP) in PBS was used, and the images were collected without the removal of CCCP. Cells were imaged using an EVOS M5000 fluorescence microscope (ThermoFisher, Waltham, MA) with a green fluorescence protein (GFP) filter cube (ex. 457–487/em. 502–538) for the green monomer fluorescence and a Texas red filter cube (ex. 542–583/em. 604–644) for the red J-aggregate fluorescence. The cellular images were analyzed using ImageJ (NIH) and the corrected total cellular fluorescence (CTCF) was calculated using the following formula:

CTCF = Integrated density – (area of cell × mean fluorescence of background reading)

For each replicate, the average red/green fluorescence was determined using at least 8 independent cells and was normalized to the untreated control cells ($[red]/[green]_{control}$ = 1). Data are reported as the average of three independent trials ± SD.

Whole Cell Uptake in HeLa Cells

Approximately 10^6 HeLa cells were seeded in 6-well plates. On the day of the experiment, the culture media was removed, and the cells were treated with fresh media supplemented with 0 or 50 µM complex and incubated for 3 h at 37 °C. The culture media was then removed, and the adherent cells were washed with 3 × 1 mL PBS and detached with 0.05% trypsin + 0.53 mM ethylenediaminetetraacetic acid (EDTA; Corning Life Sciences). The detached cells were pelleted by centrifugation (800 × *g* for 10 min), and

the supernatant was removed. The cell pellet was resuspended in PBS and pelleted again by centrifugation. This process was repeated twice more, and the resulting washed cell pellet was resuspended in ice cold lysis buffer (1% w/v 3-[3-(cholamidopropyl)dimethylammonio]-1-propanesulfonate (CHAPS), 5 mM EDTA, 50 mM tris(hydroxymethyl)aminomethane (Tris) and 100 mM NaCl; pH 7.4). The suspension was vortexed for 30 s and incubated on ice for 45 min. The cell lysate was then centrifuged to remove any precipitated debris and the supernatant was transferred to a clean tube prior to analysis. For solutions treated with any Ru containing compounds, the Ru content was determined using GFAAS and was normalized to the protein content of the sample, which was determined using the bicinchoninic acid (BCA) assay kit following manufacturer instructions (ThermoFisher). For solutions treated with any Os containing compounds, the 200 µL of the cell lysate solutions were immediately diluted to a total of 5 mL in stabilizing solution (500 µM each of ascorbic acid, thiourea, and EDTA).[4] Samples were kept at room temperature until analysis and were analyzed for Os content with ICP-MS within 7 days of preparation. The total Os content was normalized against the protein content of the sample using the BCA assay kit. Results are reported as the mean mass ratio of Ru or Os to protein content (pg $µg^{-1}$) in each sample ± SD.

Whole Cell Uptake in Primary Cortical Neurons

Cortical neuron cultures were seeded at a density of 200,000 cells/well in 24-well plates for uptake studies. After 12 d in culture, neurons were treated with **1** (0, 3, or 10 µM) or **2** (0, 3, or 10 µM) in the presence and absence of two equiv CB[7] (0, 6, or 20 µM) for 24 h. The media was then removed, and cells were washed with 3 × 500 µL cold PBS before the addition of 75 µL RIPA lysis buffer to each well. Plates were then placed on an orbital shaker for 30 min. Cell lysates were collected and centrifuged at 13,000 rpm for 15 min. The protein concentration of the cell lysates was determined using the BCA assay as mentioned previously. Samples were then stored at –80 °C for no longer than one week before analysis of Ru or Os content via GFAAS or ICP-MS, respectively. Results are reported as the mean mass ratio of Ru or Os to protein content (pg $µg^{-1}$) in each sample ± SD.

Mitochondrial Isolation

Isolation of mitochondria in HeLa cells was performed using protocols previously reported. Approximately 10^6 HeLa cells were seeded in 10 cm^2 culture dishes and allowed to adhere overnight. Each replicate consisted of cells combined from two 10 cm^2 dishes. The next day, cells were treated with 50 µM of **1** ± 100 µM CB[7] in normal culture media for 24 h at 37 °C. The media was then removed, cells were washed with 3 × 3 mL room temperature PBS and harvested with trypsin. The cells were collected and pelleted by centrifugation (800 × *g*) for 10 min and resuspended in 500 µL ice cold mitochondrial isolation buffer (pH 7.4) containing 200 mM mannitol, 68 mM sucrose, 50 mM piperazine-*N,N′*-bis(2-ethanesulfonic acid), 50 mM KCl, 5 mM ethylene glycol-bis-(β-aminoethylether)-*N,N,N′,N′*-tetraacetic acid (EGTA), 2 mM $MgCl_2$, 1 mM dithiothreitol, and 1:500 v/v protease inhibitor cocktail. The cell suspension was then incubated on ice for 20 min before it was homogenized through a 25-gauge needle using a 1 mL syringe with 40 passes. The homogenized suspension was centrifuged (150 × *g*) for 5 min, and the supernatant was transferred to a clean tube and centrifuged (14,000 × *g*) for 10 min

to pellet the mitochondrial fraction. The supernatant from this step was kept as the cytosolic fraction, while the mitochondrial pellet was suspended in 300 μL RIPA lysis buffer and vortexed at the highest setting for 30 s. The Ru content was determined as described previously and was normalized to the protein content using the BCA assay as described above. Results are reported as the mean mass ratio of Ru to protein content (pg μg^{-1}) in each sample ± SD.

Mitochondrial Ca^{2+} Uptake in Permeabilized Cells

HEK293T cells were grown to near confluency in 10 cm^2 dishes and harvested with trypsin. The cells were pelleted by centrifugation (800 × *g*) and resuspended in ice cold PBS supplemented with 5 mM EDTA (pH 7.4). This cell suspension was counted using trypan blue, and the cells were pelleted by centrifugation (800 × *g*) for 5 min and resuspended in ice cold high KCl solution (125 mM KCl, 20 mM 4-(2-hydroxyethyl)-1-piperazineethanesulfonic acid (HEPES), 2 mM K_2HPO_4, 5 mM glutamate, 5 mM malate, 1 mM $MgCl_2$; pH 7.2) supplemented with 80 μM digitonin and 1 μM thapsigargin. The final solution contained <0.1% DMSO from the original digitonin and thapsigargin stocks. The cells were incubated on ice for 15 min and centrifuged (200 × *g*) for 10 min at 4 °C. Finally, the permeabilized cells were resuspended in high KCl solution containing 1 μM Calcium Green 5N (ThermoFisher, Waltham, MA) and 2 mM succinate to a final density of 1 × 10^7 cells mL^{-1}. For each experiment, 100 μL of the cell suspension with the desired concentration of the test complex were placed in each well of a black-walled 96-well plate. The background fluorescence of each well was recorded for 60 s prior to the addition of 20 μM $CaCl_2$. The change in fluorescence of the dye (ex. 488/em. 528) in response to Ca^{2+} was recorded every 5 s for at least 120 s or until the fluorescence returned to baseline. The $_mCa^{2+}$ uptake rate was calculated as the time constant of the exponential fit of the decay in the fluorescence response curve. The Ca^{2+} uptake rate of treated cells was normalized to that of untreated cells (0% inhibition), and each replicate was performed using independently prepared cell suspensions to account for differences in cell count. A BCA assay was performed on each cell suspension for every experiment, giving an average protein content of 1200 μg mL^{-1} each time. The Hill Equation was used to determine the IC_{50} of MCU inhibition. Data are presented as the average of three independent biological replicates ± SD.

Mitochondrial Ca^{2+} Uptake in Intact HeLa Cells using Rhod2-AM

Approximately 5 × 10^4 HeLa cells were seeded in an 8-well μ-slide (Ibidi USA, Inc., Fitchburg, WI) and incubated overnight at 37 °C. The following day, cells were treated with the desired complex in DMEM supplemented with 10% FBS for 1 h at 37 °C. The culture media was removed, and the cells were washed with 100 μL PBS before the cells were incubated in the dark for 30 min at room temperature in extracellular medium (ECM; 135 mM NaCl, 20 mM HEPES, 5 mM KCl, 1 mM $MgCl_2$, 1 mM $CaCl_2$) supplemented with 10 mM glucose, 3.2 mg mL^{-1} bovine serum albumin (BSA), 0.003% Pluronic F127, and 2 μM Rhod2-AM (Molecular Probes). The dye-containing ECM was removed, cells were washed with 100 μL ECM, and cells were treated with fresh ECM supplemented with 10 mM glucose and 3.2 mg mL^{-1} BSA followed by further incubation in the dark for 30 min at room temperature. The buffer was then removed, cells were washed with 100 μL ECM, and cells were treated with fresh ECM supplemented with 10 mM glucose and 3.2 mg

mL^{-1} BSA. The cells were then incubated for 15 min at 37 °C before imaging using a Zeiss LSM i710 confocal microscope with a 40× water objective at an excitation wavelength of 561 nm and an emission window of 568-712 nm. After ~30 s of baseline recording, histamine (final concentration of 100 μM) was added to the dish and fluorescence images were collected every 3 s to monitor $_{m}Ca^{2+}$ uptake. Images were analyzed and quantified using ImageJ and the CTCF was calculated. The average of at least six individual cells were used to determine the average CTCF for each replicate. Results are reported as the average of two independent replicates ± SD.

Cell Viability after OGD

Primary cortical neuron cultures were seeded at a density of 150,000 cells/well in 48-well plates for cell viability studies. After 10–12 d, neurons were left untreated (control) or treated with the desired complex in the presence or absence of two equiv CB[7] for 3 h. Cells were then subjected to 90 min OGD. For OGD, culture media was removed and 300 μL of glucose-free balanced salt-solution (GBSS) containing corresponding concentrations of compound was added to wells. Culture plates were then placed in an airtight chamber flushed with a gas mixture of 90% nitrogen, 5% carbon dioxide, and 5% hydrogen for 3 min to remove any oxygen from the chamber. The chamber was then placed in a 37 °C incubator for the remainder of the OGD treatment (90 min total). After the OGD treatment, the GBSS was removed, and 500 μL fresh culture media containing corresponding concentrations of compound was added back to the wells. Cell viability was measured 24 h after OGD treatment using the MTT assay.

Measurement of Seizure-Like Behavior

Adult male C57/Bl6 mice (10–12 weeks old) were administered an i.p. injection of 10 mg kg^{-1} of **1** or a premixed solution of 10 mg kg^{-1} **1** and 20 mg kg^{-1} CB[7] (n = 4 for each treatment). Animals were then observed for approximately 100 min, and the duration of time each animal exhibited seizures was recorded.[11] Over time, seizure activity changed from shorter (30–120 s) mild seizures to longer (8–10 min) more severe seizures. All mice were euthanized with an overdose injection of sodium pentobarbital (150 mg kg^{-1}, i.p.) 2.5 h after the start of the experiment.[11]

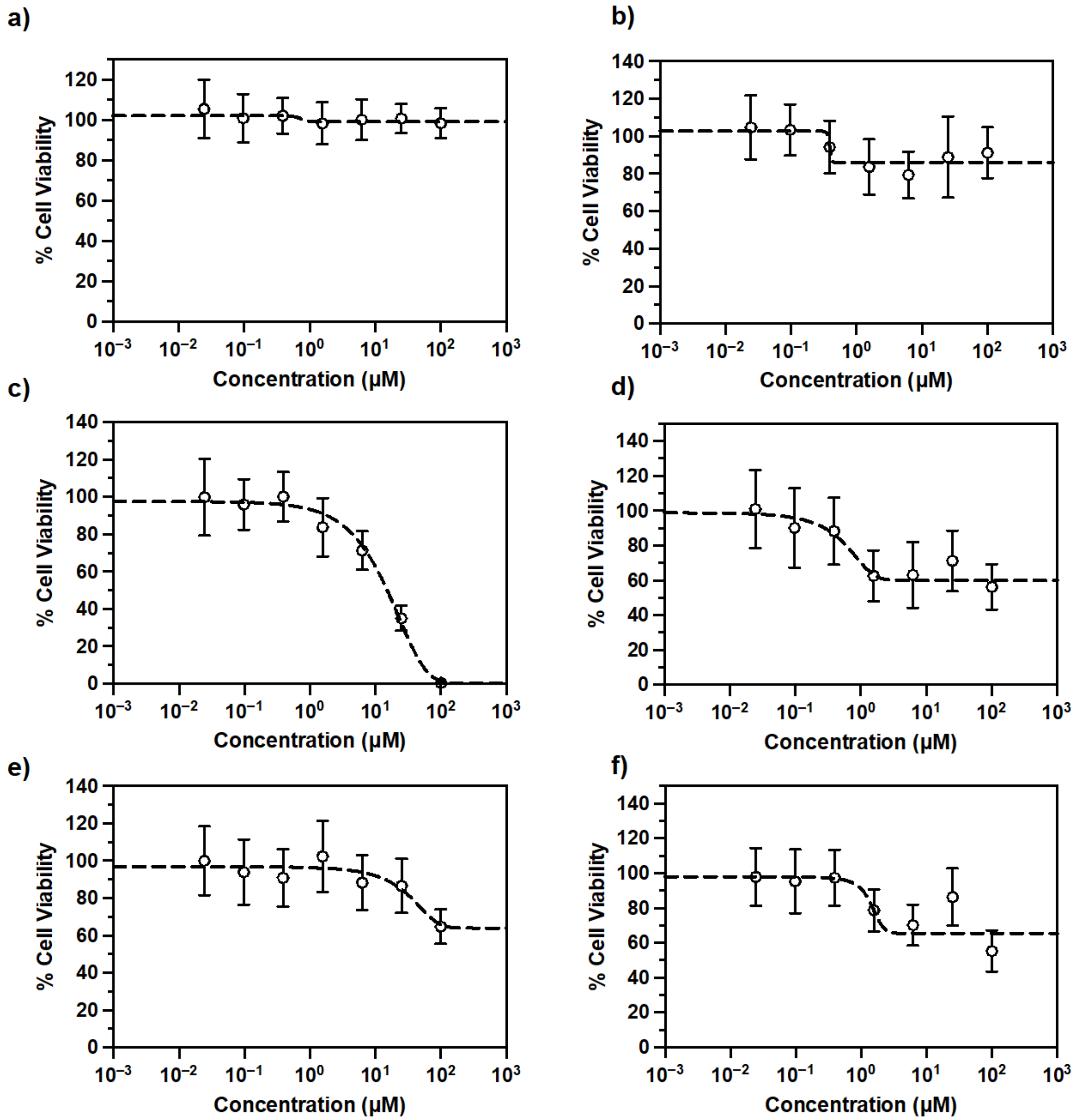

Figure S27. Cytotoxicity dose response curves in HeLa cells after 48 h for a) NaOAd, b) NaOAd + CB[7], c) **1**, d) **1** + CB[7], e) **2**, and f) **2** + CB[7]. Only compound **1** shows cytotoxicity with an IC_{50} of 21.9 ± 2.0 μM. All other treatments showed greater than 90% cell viability at concentrations less than 100 μM.

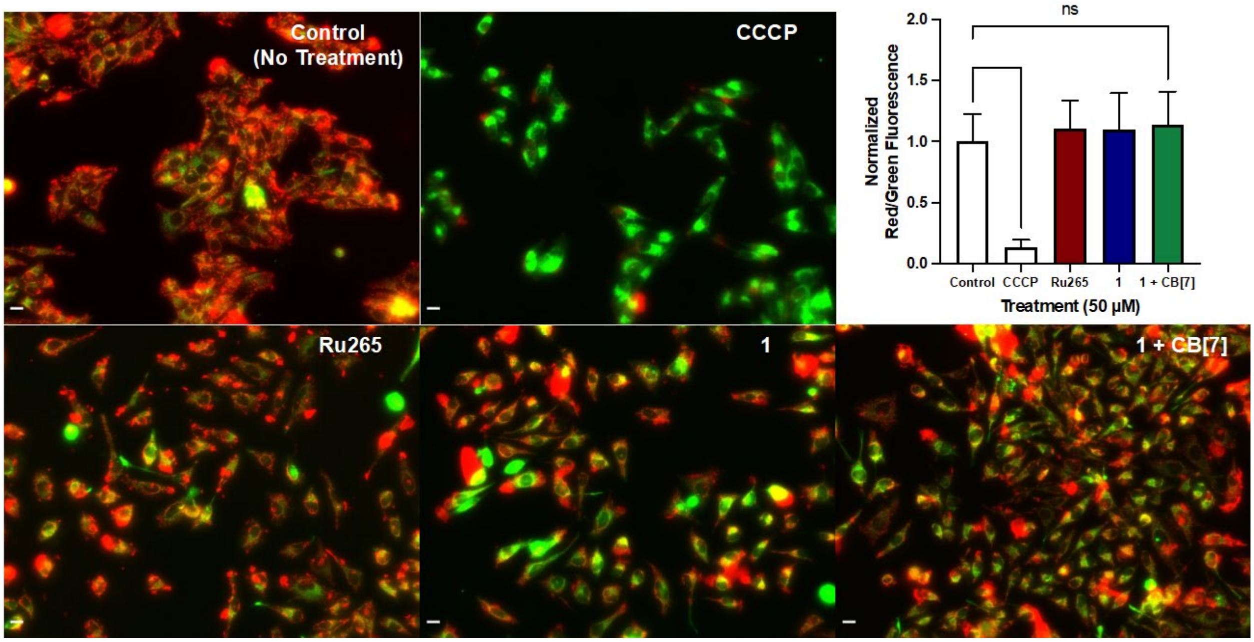

Figure S28. Fluorescent microscopy images of HeLa cells treated with JC-1 dye and 50 μM of CCCP, Ru265, and **1** ± CB[7] (scale bar = 10 μm). Top right: normalized red/green fluorescence intensity of all images.

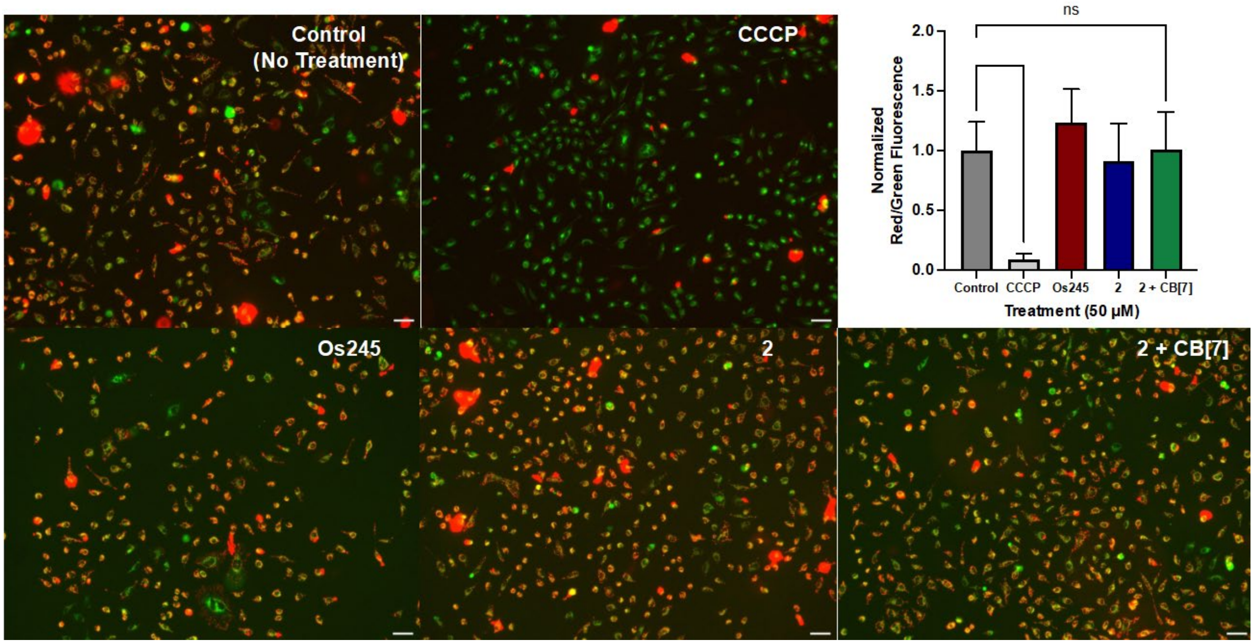

Figure S29. Fluorescent microscopy images of HeLa cells treated with JC-1 dye and 50 μM of CCCP, Os245, and **2** ± CB[7] (scale bar = 50 μm). Top right: normalized red/green fluorescence intensity of all images.

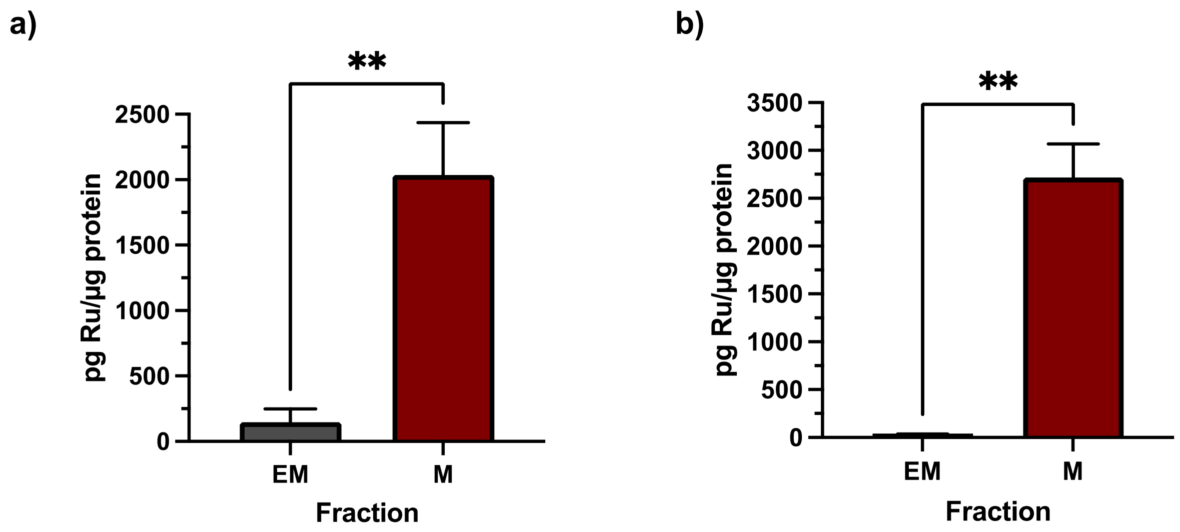

Figure S30. Comparison of the Ru concentration in the extramitochondrial (EM) and mitochondrial (M) fractions of HeLa cells treated with a) 50 µM **1** and b) 50 µM **1** + 100 µM CB[7]. Cells were treated with the desired complex for 24 h at 37 °C. Ru concentration was normalized to protein content using the BCA assay. Data are the mean of three trials ± SD (n = 3), **p < 0.01.

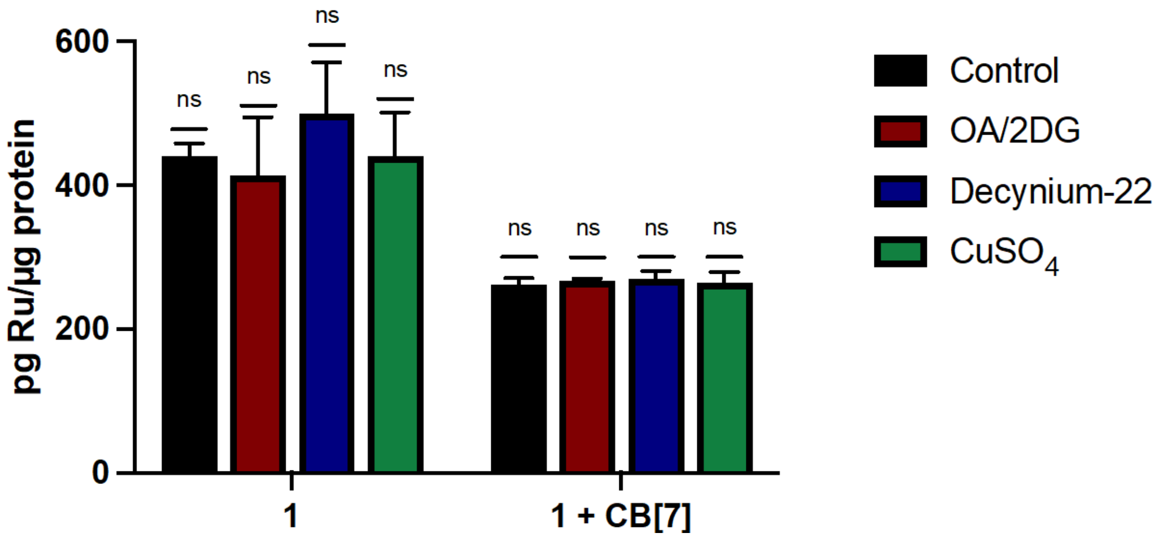

Figure S31. Cellular uptake of 50 µM **1** ± CB[7] (3 h dose) under the following conditions: 37°C (black), 50 mM 2-deoxy-D-glucose and 5 mM oligomycin A (red), 1 µM decynium-22 (blue), and 200 µM $CuSO_4$ (green). Ru concentration was normalized to the protein content using the BCA assay. Data are presented as the mean ± SD. *ns* = not significant.

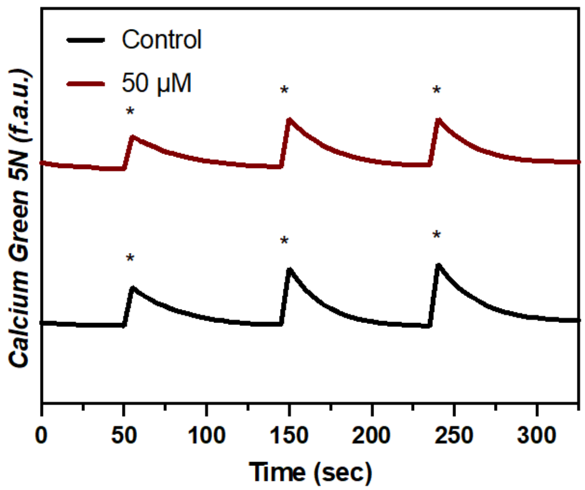

Figure S32. $_m$Ca^{2+} uptake transient curves in permeabilized HEK293T cells (1 × 10^7 cells mL^{-1}) for NaOAd (0 or 50 µM). Additions of 20 µM $CaCl_2$ are indicated with an asterisk (*).

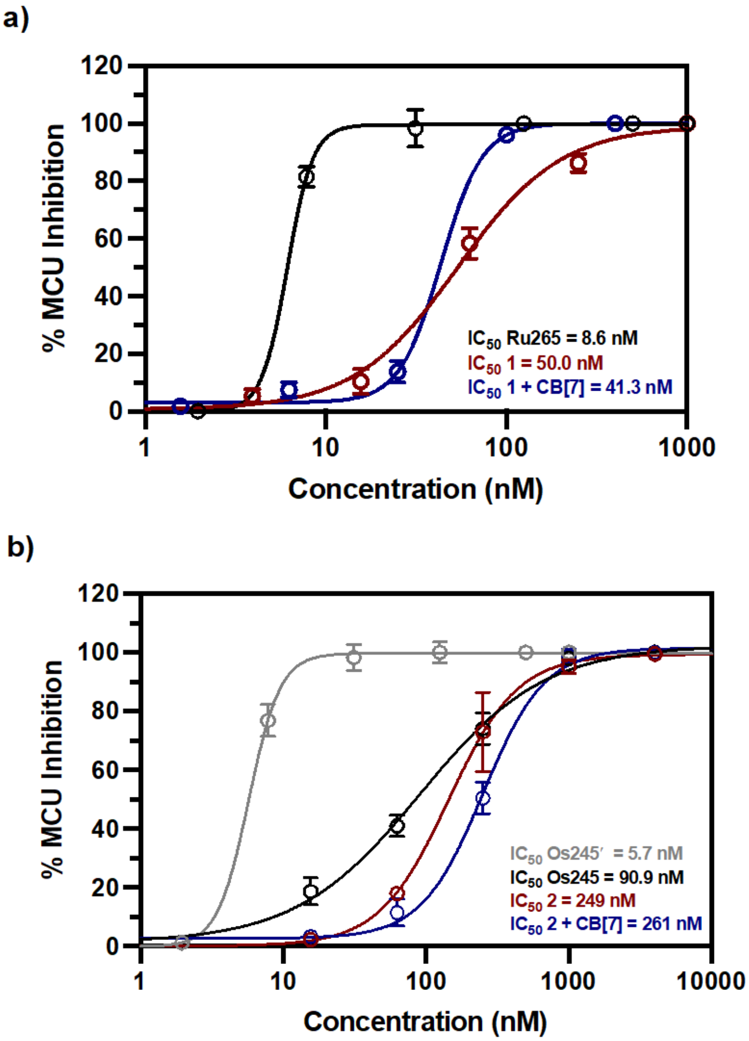

Figure S33. Dose response curves in permeabilized HEK293T cells (1 × 10^7 cells mL^{-1}) for a) Ru265, **1**, and **1** + CB[7] and b) Os245, Os245′, **2**, and **2** + CB[7]

6. References

1 J. J. Woods, N. Nemani, S. Shanmughapriya, A. Kumar, M. Zhang, S. R. Nathan, M. Thomas, E. Carvalho, K. Ramachandran, S. Srikantan, P. B. Stathopulos, J. J. Wilson, M. Madesh, A Selective and Cell-Permeable Mitochondrial Calcium Uniporter (MCU) Inhibitor Preserves Mitochondrial Bioenergetics after Hypoxia/Reoxygenation Injury, *ACS Cent. Sci.*, 2019, **5**, 153–166.

2 J. J. Woods, J. Lovett, B. Lai, H. H. Harris, J. J. Wilson, Redox Stability Controls the Cellular Uptake and Activity of Ruthenium-Based Inhibitors of the Mitochondrial Calcium Uniporter (MCU), *Angew. Chem., Int. Ed.*, 2020, **59**, 6482–6491.

3 J. J. Woods, R. J. Novorolsky, N. P. Bigham, G. S. Robertson, J. J. Wilson, Dinuclear Nitrido-Bridged Osmium Complexes Inhibit the Mitochondrial Calcium Uniporter and Protect Cortical Neurons against Lethal Oxygen–Glucose Deprivation, *RSC Chem. Biol.*, 2023, **4**, 84–93.

4 M. H. M. Klose, M. Hejl, P. Heffeter, M. A. Jakupec, S. M. Meier-Menches, W. Berger, B. K. Keppler, Post-Digestion Stabilization of Osmium Enables Quantification by ICP-MS in Cell Culture and Tissue, *Analyst*, 2017, **142**, 2327–2332.

5 A. C. Garcia, L. N. Zakharov, M. D. Pluth, Supramolecular Activation of S_8 by Cucurbiturils in Water and Mechanism of Reduction to H_2S by Thiols: Insights into Biological Sulfane Sulfur Trafficking, *J. Am. Chem. Soc.*, 2022, **144**, 15324–15332.

6 G. M. Sheldrick, A Short History of SHELX, *Acta. Crystallogr., Sect. A: Found. Crystallogr.*, 2008, **64**, 112–122.

7 G. M. Sheldrick, Crystal Structure Refinement with SHELXL, *Acta Crystallogr. Sect. C Struct. Chem.*, 2015, **71**, 3–8.

8 P. Müller, Practical Suggestions for Better Crystal Structures, *Crystallogr. Rev.*, 2009, **15**, 57–83.

9 R. O’Brien, N. Markova, G. A. Holdgate, Thermodynamics in Drug Discovery, in: Appl. Biophys. Drug Discovery, John Wiley & Sons, Ltd, 2017,: pp. 7–28.

10 N. E. Grossoehme, A. M. Spuches, D. E. Wilcox, Application of Isothermal Titration Calorimetry in Bioinorganic Chemistry, *J. Biol. Inorg. Chem.*, 2010, **15**, 1183–1191.

11 R. J. Novorolsky, M. Nichols, J. S. Kim, E. V. Pavlov, J. J. Woods, J. J. Wilson, G. S. Robertson, The Cell-Permeable Mitochondrial Calcium Uniporter Inhibitor Ru265 Preserves Cortical Neuron Respiration after Lethal Oxygen Glucose Deprivation and Reduces Hypoxic/Ischemic Brain Injury, *J. Cereb. Blood Flow Metab.*, 2020, **40**, 1172–1181.
